# An Accessible Method to Improve the Stability and Reusability of Porcine Pancreatic α-Amylase via Immobilization in Gellan-Based Hydrogel Particles Obtained by Ionic Cross-Linking with Mg^2+^ Ions

**DOI:** 10.3390/molecules28124695

**Published:** 2023-06-11

**Authors:** Camelia Elena Tincu (Iurciuc), Brahim Bouhadiba, Leonard Ionut Atanase, Corneliu Sergiu Stan, Marcel Popa, Lăcrămioara Ochiuz

**Affiliations:** 1 Department of Natural and Synthetic Polymers, “Cristofor Simionescu” Faculty of Chemical Engineering and Protection of the Environment, “Gheorghe Asachi” Technical University, 73, Prof. Dr. Docent Dimitrie Mangeron Street, 700050 Iași, Romania; camelia_tincu83@yahoo.com (C.E.T.);; 2 Department of Pharmaceutical Technology, Faculty of Pharmacy, “Grigore T. Popa” University of Medicine and Pharmacy, 16, University Street, 700115 Iaşi, Romania; 3 Laboratory of Engineering of Industrial Safety and Sustainable Development LISIDD, Institute of Maintenance and Industrial Safety, University of Oran 2, Mohammed Benahmed, Oran 31000, Algeria; 4 Faculty of Dental Medicine, “Apollonia” University of Iasi, 11, Pacurari Street, 700511 Iași, Romania; 5 Academy of Romanian Scientists, 050045 Bucharest, Romania

**Keywords:** α-amylase immobilized, gellan hydrogel particles, magnesium acetate, ionic cross-linking, hydrolysis

## Abstract

Amylase is an enzyme used to hydrolyze starch in order to obtain different products that are mainly used in the food industry. The results reported in this article refer to the immobilization of α-amylase in gellan hydrogel particles ionically cross-linked with Mg^2+^ ions. The obtained hydrogel particles were characterized physicochemically and morphologically. Their enzymatic activity was tested using starch as a substrate in several hydrolytic cycles. The results showed that the properties of the particles are influenced by the degree of cross-linking and the amount of immobilized α-amylase enzyme. The temperature and pH at which the immobilized enzyme activity is maximum were T = 60 °C and pH = 5.6. The enzymatic activity and affinity of the enzyme to the substrate depend on the particle type, and this decreases for particles with a higher cross-linking degree owing to the slow diffusion of the enzyme molecules inside the polymer’s network. By immobilization, α-amylase is protected from environmental factors, and the obtained particles can be quickly recovered from the hydrolysis medium, thus being able to be reused in repeated hydrolytic cycles (at least 11 cycles) without a substantial decrease in enzymatic activity. Moreover, α-amylase immobilized in gellan particles can be reactivated via treatment with a more acidic medium.

## 1. Introduction

Enzymes are biological catalysts that can reduce the activation energy of some chemical reactions, and the entire biotechnological field depends on their activity [1].

Different types of enzymes are used on an industrial scale, such as in the food and beverage, pharmaceutical, detergent, motor fuel (bioethanol), and leather industries. Biocatalysts used in industry are disposable and thus impose additional costs in terms of the supply of new enzymes [2,3].

Most isolated enzymes need to be stable for their use in industrial conditions. In general, the enzymes are water-soluble and, therefore, difficult to separate from reactants or reaction products and can only be used in one enzymatic cycle. The enzyme is difficult to remove from the reaction medium and constitutes an impurity in the final products. Its elimination by different methods is required, which complicates the technologies that use such catalysts [4,5].

During the process of starch hydrolysis, the stability of free α-amylases can often be compromised due to various factors such as changes in viscosity, pH, temperature, and osmotic pressure [6,7,8,9,10]. Efforts have been made to enhance the effectiveness of α-amylase, as the existing approach of utilizing a high enzyme concentration is not financially viable. Various methods have been explored to address this limitation [11,12], including developing thermostable enzymes and protein modifications that have resulted in several commercial products [13,14]. These disadvantages can also be partially or totally eliminated by immobilizing the enzyme using different methods. These methods offer five key advantages: (a) the effective recovery and reusability of the enzyme; (b) the easy separation of the enzyme from the reaction mixture; (c) the uncomplicated design and performance control of bioreactors; (d) increased enzyme activity; (e) improved catalytic features, like stability and specificity (selectivity) [15,16,17,18,19,20].

The soluble enzymes must be immobilized to ensure long-term reuse in industrial reactors. In addition, it is crucial to enhance specific essential enzyme properties, including stability, activity, selectivity to unnatural substrates, and sensitivity to reaction products [21]. The selection of the appropriate immobilization method is critical for achieving optimal enzyme immobilization. This process is essential for using enzymes as industrial biocatalysts and provides a straightforward solution for overcoming the challenges of solubility and stability [22].

In order to reuse enzymes efficiently, their catalytic properties must be maintained, and the support material used for immobilization should be stable. Immobilization is a process that can enhance enzyme stability if it is properly performed. However, it is important to be careful with random immobilization techniques as they can also lead to a decrease in the catalytic activity and stability of the enzymes. Immobilization techniques must be used to stabilize enzymes against inhibitors, preventing aggregation, autolysis, or proteolysis [23,24,25,26]. The immobilization of α-amylase can also modify the enzyme selectivity and specificity, reduce inhibitions, or be coupled to purification [15,27,28,29,30].

Coating immobilized enzymes with hydrophilic polymers help prevent the inactivation and inhibitory effects determined by the interactions with organic solvents in industrial processes [31]. When selecting the support for enzyme immobilization, it is crucial to consider the characteristics of the enzyme and choose the appropriate functional groups and immobilization conditions. Supports like agarose beads [32], zeolites [33], porous glass [34], and epoxy resins [35] offer a large surface area for enzyme-support interactions. However, it is important to note that glutaraldehyde chemistry, while being a popular technique, determines the chemical modification of the entire enzyme surface [36,37]. When selecting the ideal conditions for enzyme immobilization, it is crucial to consider various factors, such as the reaction time, pH value, temperature, buffer solution employed, and any of the potential inhibitors present during the reaction [38].

Enzymes need to have high specificity for their natural substrate, but when they are immobilized, this specificity can decrease significantly [24]. Potato starch, corn starch, and dye-labeled starch are commonly utilized substrates when amylase activity is determined. It has been observed that pancreatic amylase exhibits a higher affinity for cornstarch when used as the substrate than for salivary amylase. The variation in amylase activity can also be illustrated by determining the K_m_ and V_max_ values of immobilized and free pancreatic amylase [39,40]. The immobilization process can cause distortion in the protein’s active site, leading to the reduced mobility of functional groups and decreased substrate affinity [41]. Mild immobilization techniques are necessary to maintain the enzyme’s natural properties and prevent unwanted restrictions from enzyme mobility or distortions in the protein’s active sites [24,42].

There are several ways in which immobilization can effectively solve problems related to inhibition. Additionally, successful strategies have included enzyme co-aggregation with hydrophilic polymers and entrapment in hydrophilic particles. Immobilization is a reliable solution for preventing enzyme inhibition caused by inhibitors targeting the different active sites of its structure [31,43]. Enzyme complexation with polysaccharides in order to form coacervates is a simple, green, and efficient way to immobilize and stabilize enzymes without organic solvents and any subsequent purification. It was shown that α-amylase with λ-carrageenan complexes did not dissociate at pH 1.0–2.0 and helped protect α-amylase against the severe acidic environment, and almost 70% of enzyme activity was preserved [44].

Encapsulation, adsorption, cross-linking, and covalent attachment are the most common enzyme immobilization methods. The immobilized enzymes have different applications and can be used in several hydrolytic cycles, thus reducing production costs and improving catalytic activity by increasing its stability [45,46]. The limitations of enzyme immobilization are related to its slow diffusion, conformational changes (adsorption method), loss of enzyme activity, mass transfer limitation, low enzyme loading (encapsulation method), low reproducibility, handling difficulties (cross-linking method), difficulty in preparation, and loss of enzyme activity due to reactions with toxic cross-linking reagents (covalent attachment method) [47].

Enzyme leakage could occur using different types of immobilization techniques but could be minimized by coating the surface of the immobilized biocatalysts with a polymer, such as poly(ethyleneimine) (PEI) [48,49,50]. The native structure of the immobilized enzyme remains intact, while the carrier can be designed to provide an optimal environment for the enzyme [48]. Enzyme leakage can be avoided using the encapsulation method in biopolymer-based hydrogel particles by adjusting the cross-linking degree.

During the encapsulation, the enzymes are immobilized in the porous synthetic or natural polymeric matrix (such as a hydrogel), and the substrate (starch) freely diffuses through it. It represents an accessible and rapid method that requires mild conditions and protects the enzyme from mechanical shear, hydrophobic solvents, and gas bubbles. However, it has some disadvantages, such as limitations in mass transfer, enzyme loading at a low level, and loss of enzyme activity [51,52]. Polymer porosity, surface functionality, network structure, and particle size can be modified to solve these issues [47].

In the case of enzyme encapsulation in a hydrogel matrix by ionic cross-linking, the cross-linking degree increases with the concentration of metal ions (divalent or trivalent). The polymer network meshes and porosity become smaller with increasing the cross-linking degree. The substrate diffusion into the polymer matrix is difficult to perform, and enzymatic activity can decrease. However, the functional groups of anionic polysaccharides can interact with enzyme amino groups from lysine residues, forming polyelectrolyte complexes that retain enzymes within the hydrogel. Cross-linked polymer matrices can increase immobilized enzyme stability, but a balance is needed to maintain its catalytic activity [48,53,54,55].

Hydrolases are enzymes with essential applications in the food industry. Among the most important representatives of this class is α-amylase, which produces the hydrolysis of α-1,4 glycosidic bonds in polysaccharides with a polymerization degree at least equal to 3. Amylose, a polysaccharide in the composition of starch, is cleaved under the action of α-amylase to oligosaccharides with a lower polymerization degree, called dextrins. However, α-amylases cannot cleave α-1,6-glucosidic bonds in amylopectin, the second component of starch [56,57].

The hydrolysis reactions occur at an optimal pH, and the reaction temperature depends on the type of α-amylase used [58,59]. The α-amylase produced by various bacteria such as *Bacillus licheniformis*, *Bacillus amyloliquefaciens*, *Bacillus subtilis*, *Aspergillus oryzae*, and *Rhizopus* sp. are widely used in different industrial sectors, including the production of detergents, paper, textiles, and the food industry [60].

The immobilization influences kinetic parameters, such as the maximum reaction rate (V_max_) and the Michaelis constant (K_m_), which have higher values than those for the free enzyme and, consequently, a lower affinity for the substrates [47].

The α-amylase from various sources was immobilized on different matrices, such as a chitin-bentonite hybrid matrix, an amidrazone acrylic fabric, a covalently cross-linked chitosan matrix, an alginate ionically cross-linked matrix, and nano-sized zeolite-based materials. Immobilization improved α-amylase stability (measured by t1/2 half-life), thermal stability, pH stability, starch affinity, and storage stability, and the enzyme could be reused in several hydrolytic cycles, making it suitable for various industrial applications. Nano-zeolite immobilization was found to be particularly effective in increasing α-amylase stability against denaturation and storage [43,61,62].

α-amylase, an endoenzyme from the porcine pancreas, is a glycoprotein that consists of a single polypeptide chain of approximately 475 residues that contains two SH groups and four disulfide bridges and forms tight bonds with calcium ions [63].

A specific problem in the design of a biocatalyst is whether the enzyme is multimeric or monomeric. The inactivation of these enzymes begins, in many cases, by dissociating the enzyme into its subunits. There are various types of multimeric enzymes, such as aldolases, catalases, dehydrogenases, galactosidases, and oxidases [50].

Even if this is not relevant for enzyme stability, the release of enzyme subunits can contaminate the final product. However, in this case, the immobilization-stabilization strategies should take into account the enzyme’s multimeric nature and target not only the attachment of the protein but also the subunit attachment.

There is a strategy that involves immobilizing multimeric enzymes in polymer matrices and cross-linking the enzyme subunits after immobilization, which has been recommended for various enzyme types. Another method for multimeric enzyme immobilization is through multipoint covalent attachment to different supports [48]. One of the most recent and interesting observations about porcine pancreatic α-amylase is its processive nature [64,65]. This means that the enzyme does not detach from its polymeric substrate after releasing the product but binds randomly at a point on the polysaccharide chain, cleaving it and releasing maltose or maltotriose units over time. Typically, this type of processivity is associated with toroidal enzyme complexes, such as DNA or RNA polymerase [66]. However, unless α-amylase functions as a multimer rather than a monomer, a feature that has not been investigated in detail, its processivity may not arise from the formation of a toroidal complex but rather from other properties of the enzyme [67]. Porcine pancreatic α-amylase is synthesized as a single polypeptide chain, and previous studies have confirmed that α-amylase from the porcine pancreas is a monomeric enzyme but undergoes some proteolysis to yield smaller associated fragments that retain their activity [67,68].

Pancreatic α-amylase is mainly responsible for the in vivo degradation of starch. In humans, starch digestion is initiated by salivary amylase in the mouth. Starch is degraded into oligomers that are further degraded by pancreatic α-amylase into maltose, maltotriose, and low-molecular-weight maltooligosaccharides in the small intestine. Porcine pancreatic amylase was separated into two isoforms, and their molecular weight was 55 kDa. α-amylases are metalloenzymes containing at least one Ca^2+^ ion per enzyme molecule, which is essential for their activity and stability. Calcium with a concentration of 1–20 ppm is required to maintain the structural integrity of amylases. The removal of calcium ions causes a decrease in thermostability and enzyme activity [69].

Previous research shows that α-amylase enzyme activity increases in the presence of magnesium and potassium and decreases in the presence of high levels of copper and calcium ions [70,71,72]. Magnesium and manganese have also been reported as co-factors stimulating α-amylase activity [73].

The literature reports many examples of α-amylase immobilized on different supports. Dextrin and dextran as substrates for α-amylase are antithrombotic agents (antiplatelet), which are agents used to decrease blood viscosity and increase blood vessel volume in hypovolemic patients [47]. By conjugating polymers with different bioactive molecules, specific tissues are protected from their adverse action, and the conjugated biomolecules are also protected from degradation. Exposure of the conjugate to α-amylase causes the controlled and sustained release of bioactive molecules, inducing EGFR phosphorylation in cells [74,75]. Some commercial products are made using this concept, namely dextrin-colistin and tilmanocept [76] α-amylase replacement therapy, which is an appropriate therapeutic method in pancreatic insufficiency disorders such as cystic fibrosis. In these patients, α-amylase is not secreted in adequate concentrations [77].

Gellan is an anionic polysaccharide with a high molecular weight produced in an aerobic environment by *Aeromonas (Pseudomonas) elodea.* Due to its biocompatible and biodegradable properties, gellan is used in various applications, especially in the food and pharmaceutical industries. Gellan shows excellent gelling properties, and the gels obtained are strong, translucent, and stable at low pH. Low concentrations of gellan can result in a resistant hydrogel in the presence of bivalent metal ions [78,79].

Gellan allows for immobilization via ionic cross-linking with the Ca^2+^ ions of the amylase extracted from marine *Nocardiopsis* sp. *foreigner B2* [80]. In another piece of research, gellan was oxidized in the presence of NaIO_4_ and could form conjugates with α-amylase from *Bacillus licheniformis* via the reaction between the aldehyde groups from oxidized gellan and the amino groups from α-amylase [81].

In previous research, we obtained gellan-based hydrogel particles ionically cross-linked with magnesium acetate, zinc acetate, or calcium chloride using immobilized yeast cells that can be used in multiple fermentation cycles for ethanol production. Thus, it was demonstrated that the gellan matrices obtained by ionic cross-linking are stable, maintain the cellular viability of yeast cells, and can be used in several different fermentation cycles; the maximum number was 40 for the gellan particles cross-linked with magnesium acetate [82,83,84].

The results reported in the present article refer to the immobilization of α-amylase in gellan hydrogel particles, ionically cross-linked with Mg^2+^, using the extrusion of the polysaccharide solution containing the enzyme in an ionotropic gelation bath containing different concentrations of magnesium acetate.

The aim of this research was to assess the stability of α-amylase, which was immobilized in gellan particles. We also aimed to examine the activity of this immobilized biomolecule in the presence of starch.

Particles with immobilized α-amylase could have applications in treating pancreatic insufficiency, such as cystic fibrosis. Research has demonstrated the effectiveness of α-amylase in the therapy of dyspepsia, anorexia, heartburn, and postprandial distension [85]. The gellan matrix is highly resistant to stomach enzymes and acidic pH. The immobilized enzyme can be released from the polymer matrix in a controlled and sustained manner while avoiding the accumulation of a large amount of sugar in the bloodstream [86]. The presence of α-amylase triggers starch hydrolysis, which leads to the production of maltose and glucose; these monosaccharides have been widely used in pharmaceutical formulations, such as syrups.

In order to monitor the enzymatic activity of α-amylase, the colorimetric method of determining substrate consumption with iodine solution (UV-VIS spectroscopy) was used.

Although the determination of reducing sugars obtained from the starch hydrolysis reaction is typically carried out using dinitrosalicylic acid, it is a costly method that requires a significant amount of reagents and has a complex protocol [87]. According to the literature, the α-amylase activity in the samples was found to be two times higher when measured by the iodine solution test compared to the DNS method [88], probably due to the fact that there are other reaction products that can form from the hydrolysis of starch, not only maltose.

The use of α-amylase immobilized in different polymeric supports in the pharmaceutical industry is limited, and in this work, we want to establish some physicochemical characteristics of the immobilized enzyme in gellan particles in comparison to the free enzyme.

The main objective of this research is to obtain stable gellan particles containing α-amylase that have the ability to hydrolyze the starch.

Another aim of this work is to physicochemically characterize the immobilized enzyme compared to the free enzyme to establish how the enzyme’s catalytic activity is influenced after immobilization, as well as the protective role of the polymer matrix against environmental factors that can determine the modification/inhibition of enzyme activity; these latter aspects give our work a novel character.

The originality of our work lies in the immobilization of α-amylase in hydrogel particles ionically cross-linked with magnesium acetate. Mg^2+^ ions were used for cross-linking the gellan-based particles because an increase in the activity of the bioactive component immobilized in this type of particles was observed [82] compared to the studies carried out with gellan particles cross-linked with Ca^2+^ [83] or Zn^2+^ ions [84].

Particles with immobilized α-amylase were characterized physicochemically, morphologically, and from the point of view of enzymatic activity in several hydrolytic processes using starch as the substrate. Another originality element of this research is that the α-amylase-loaded gellan hydrogel particles have not been tested until now in different hydrolysis cycles, and the Michaelis–Menten parameters, such as V_max_ and K_m_, for the immobilized enzyme in these types of particles, have not yet been determined.

## 2. Results and Discussions

### 2.1. Characterization of the Free Enzyme

In order to compare the immobilized enzyme with the free enzyme, it was first necessary to characterize the latter. Free enzyme assays were performed to determine enzymatic activity as a function of starch concentration and the time required for a complete hydrolysis reaction. The rate of hydrolysis represents the amount of hydrolyzed starch (µmols)/minute.

- ***Determination of the time required for a hydrolysis reaction***

The working method is presented in Section 2.3. Figure S1 illustrates the variation of the starch hydrolysis rate with time using 200 μL of the free enzyme from the stock solution, which has a concentration of 39.22 mg/100 mL. Figure S2 shows the hydrolyzed starch formation rate variation and enzymatic activity as a function of α-amylase concentration.

It can be seen from Figure S1 that the rate of starch hydrolysis decreases over time and, after 10 min, remains almost constant. For this reason, the time required for each hydrolysis cycle was determined to be 10 min.

The measurements for time with the hydrolysis of starch are different, and this depends on both the type of enzymes and the brand of alpha-amylase. Aksoy, S et al. [89] found that the time required for a complete hydrolysis cycle was 5 min, and Guo H et al. [90] found a total hydrolysis cycle of 30 min. It was found that the difference was due to the brand. Guo H et al. used the α-amylase from Shanghai Kaiyang Biological Technology Co. (Shanghai, China), and Aksoy S et al. used the enzyme from Merck AG (Darmstadt, Germany) [6].

Additionally, it can be observed from Figure S2 that the hydrolysis rate increases with increasing α-amylase concentration. These preliminary tests on the free enzyme were necessary in order to determine the free enzyme’s activity and the Michaelis–Menten kinetics, respectively, as the K_m_ and V_max_ constants.

### 2.2. Obtaining Gellan Particles with Immobilized α-Amylase

The gellan particles with immobilized α-amylase were obtained using the ionic cross-linking method with magnesium acetate. In order to maintain the enzymatic activity of α-amylase, gellan was dissolved in an acetate buffer solution at pH 5.6 (0.05 M).

The total volume of α-amylase solution (3 mg/mL) added to the gellan solution was 1 mL. Table 1 shows the experimental program, enzymatic activity, and immobilization efficiency. Several α-amylase concentrations were initially used for immobilization, and the immobilization efficiency was determined using Equation (2) (Section 3.2.3). The Lowry method was used to determine the total amount of proteins in the supernatant. The enzymatic activity was determined using Equation (1) (Section 3.2.1). Based on the results presented in Table 1, the optimal enzyme concentration that can be immobilized in the obtained samples was determined.

The optimal temperature at which the solution containing α-amylase is added to the gellan solution before extrusion was determined. The temperatures of the gellan solution at the time of α-amylase addition were 40, 50, and 60 °C. We chose these temperatures because, at the lower values (30 °C), the gellan solution is very viscous and cannot be extruded, leading to the formation of non-spherical and dimensionally polydisperse particles. At a higher temperature (70 °C), it is possible to produce the deactivation of the enzyme [69]. Table S1 (supplementary information) shows the results obtained after testing the enzymatic activity of the particles prepared at different temperatures. It was found that the best enzymatic activity was obtained when the immobilization temperature was 60 °C.

The hydrogel particles have an average diameter of 2.5 mm in the swollen state. The stability of gellan particles with or without yeast cells immobilized and ionically cross-linked with magnesium acetate was evaluated in a previous study [82]. The hydrogel particles were found to be stable, and the activity of the bioactive principle immobilized was maintained at a high level. The results regarding the immobilization efficiency of α-amylase in gellan particles and the enzymatic activity of the immobilized enzyme in one gram of particles are shown in Table 1.

Table 1 shows that immobilization efficiency depends on the enzyme amount used for encapsulation and the cross-linking degree of the particles. Thus, the maximum enzymatic activity was obtained for the particles with an immobilization efficiency equal to 75.56 ± 5.08%, and gellan particles were prepared using a concentration of 2% magnesium acetate in the cross-linking bath and 3 mg of enzymes for immobilization. Immobilization efficiency also depends on the magnesium acetate concentration. It increases when the magnesium acetate concentration within the cross-linking bath increases, and the polymer network meshes become smaller when the cross-linking density increases. As a result, α-amylase is more firmly anchored in the polymer mesh network and cannot diffuse from the polymer matrix. According to the ANOVA statistical test, the immobilization efficiency values for the samples prepared with a 2% magnesium acetate concentration in the cross-linking bath did not show any statistical differences. The *p*-parameter value was higher than 0.05 (*p* = 0.25). However, statistical differences were observed for the immobilization efficiencies in samples A3, A4, and A5, where the *p*-parameter value was less than 0.05 (*p* = 0.036). The enzymatic activity value for sample A5 (1 g of particles) was lower than that recorded for the other types of particles. The explanation also consists of the reduced diffusion of the substrate (starch) to the encapsulated enzyme, determined by the higher cross-linking degree of the particles.

**Figure 1:**
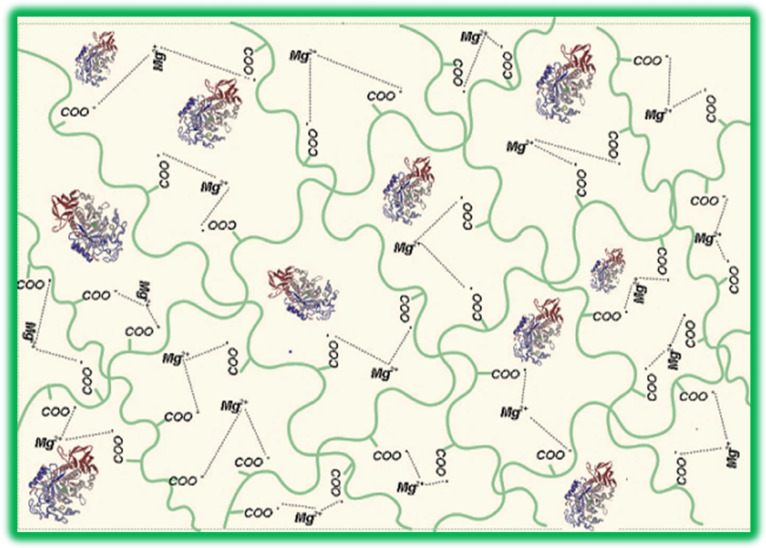
Schematic structure of α-amylase immobilized in ionically cross-linked gellan particles.

The enzymatic activity of α-amylase depends on the amount of immobilized enzyme. Table 1 shows that the particles containing 3 mg of enzyme, obtained at a concentration of 2% magnesium acetate in the cross-linking bath, have optimal immobilization efficiency and enzymatic activity. These samples were used to determine the physicochemical characteristics of the particles with immobilized α-amylase compared with non-immobilized α-amylase. Enzymatic activity decreases for particles A6, A7, and A8, which contain a higher amount of immobilized α-amylase. The concentration of 2% magnesium acetate was in excess, and the number of free carboxylic groups in gellan after ionic cross-linking decreased. The large number of free amino groups in α-amylase can no longer interact with the carboxylic groups, leading to the saturation of the polymer matrix with enzyme molecules where protein-protein interactions (steric intramolecular hindrance) could occur, which causes lower enzymatic activity [91,92]. The results obtained for the determination of enzyme activity for A4 gellan particles with immobilized α-amylase are similar to those obtained by Xiao Z et al., who obtained a value for free α-amylase (from *Aspergillus oryzae*) of 4.92 ± 0.07 and 4.71 ± 0.06 [88]. Figure 1 shows the schematic structure of gellan particles with immobilized α-amylase.

The α-amylase is immobilized in the meshes of the network formed by ionic cross-linked gellan, according to the reaction

Mg^2+^(^−^OCOCH_3_)_2_ + 2Gellan-COO^−^Na^+^ → Gellan-COO^−^Mg^2+ −^OCO-Gellan + 2Na^+^ + 2CH_3_COO^−^

### 2.3. FT-IR Spectroscopy

**Figure 2:**
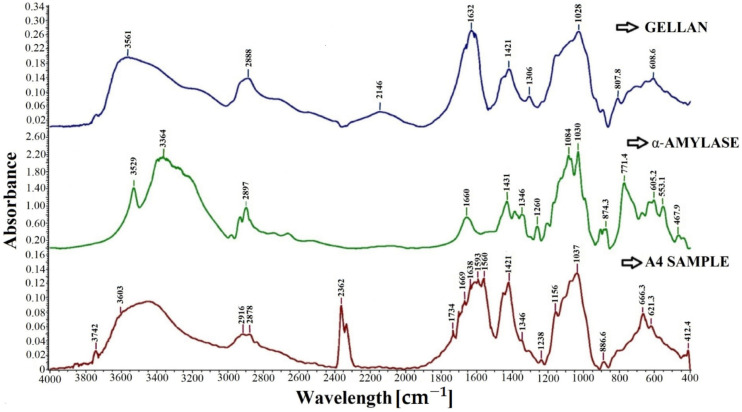
FT-IR spectra of gellan, α-amylase, and sample A4.

The FT-IR spectra, shown in Figure 2, were recorded for gellan, free α-amylase, and sample A4.

The spectrum of gellan shows an intense absorption band at about 1026 cm^−1^, attributed to C-O-C stretching and specific to the carboxylic groups in gellan. The characteristic absorption peaks at 1632 and 1421 cm^−1^ presented in the FTIR spectrum of gellan correspond to COO- asymmetric and symmetric stretching vibrations, respectively [93]. The absorption peak at 1421 cm^−1^ can be attributed to the C=O stretching vibration belonging to the carboxylic group, or it can be attributed to the CH_3_ group in the rhamnose unit. Amylase shows the typical amide bands specific to proteins. The absorption peak of the amide band at 1660 cm^−1^ could be attributed to C=O or N-H stretching vibrations.

All the peaks that are characteristic of the enzyme or gellan are found in the spectrum of sample A4. The broadband characteristic of the enzyme due to the presence of overlapping OH and NH groups present in α-amylase is evident at around 3603 cm^−1^ in the gellan particle with immobilized enzyme, i.e., the A4 sample spectrum. Meanwhile, a CH stretching vibration is observed around 2878 cm^−1^ in the spectrum of sample A4. Furthermore, the spectrum of sample A4 shows the presence of amide bands. These bands are observed at 1669 cm^−1^, corresponding to the CO group’s stretching vibration (amide I). The bending vibration of the NH group (amide II) is observed at 1560 cm^−1^, while a weak band at 1238 cm^−1^ corresponds to the stretching vibration of CN (amide III) [43]. The appearance of a broad absorption band between 1560 cm^−1^ and 1734 cm^−1^ with peaks characteristic of both gellan and α-amylase can be observed in the spectrum of sample A4. The absorption peak shift after immobilization represents a change in the structure of the protein from its native form [94]. The FT-IR spectrum of A4 particles revealed shifts in the absorption peaks characteristic of both gellan and α-amylase. The spectrum of sample A4 shows absorption peaks at 666.3 cm^−1^ and 621.3, which can be attributed to the Me-O bond [94,95,96].

The absorption peak of low intensity from 1734 cm^−1^ indicates the electrolytic dissociation of the -CH_2_COO^−^Na^+^ group and occurs especially in polyelectrolyte complexes. The literature states that this absorption band is stronger if amino group consumption intensifies [97,98].

Additionally, the peaks from 1560 cm^−1^ and 1593 cm^−1^ indicate the stretching vibrations of the N–H, C–N, and C–C groups. It may also indicate some interactions between the amino groups in α-amylase and the carboxylic groups in gellan. It is observed that the characteristic band of amino groups in α-amylase at 3529 cm^−1^ appears to shift in the spectrum of A4 particles at around 3600 cm^−1^, which may indicate some intermolecular interactions or hydrogen bond formation [99].

### 2.4. Morphology of the Particles with Immobilized α-Amylase

The same amount of α-amylase was immobilized in the particles analyzed by scanning electron microscopy, with the samples differing in the cross-linking agent concentration used in the gelation bath at 1% and 2% magnesium acetate, respectively. The samples were dried, and scanning electron microscopy photographs were taken in the particle cross-section.

**Figure 3:**
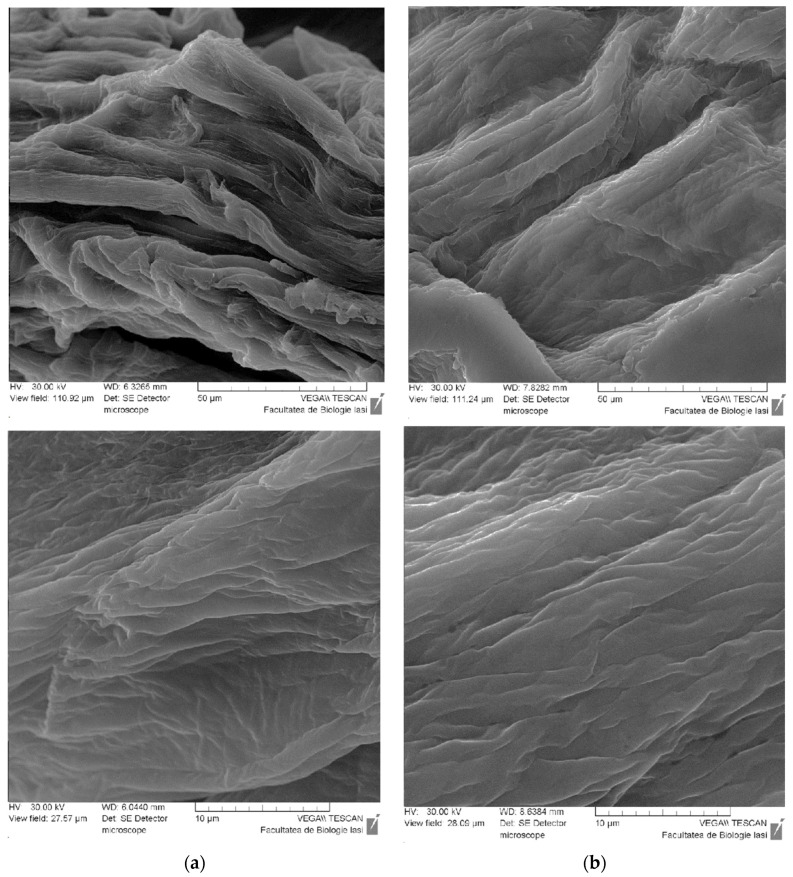
Scanning electron microscopy photographs of particles containing immobilized α-amylase cross-linked with (**a**) 1% and (**b**) 2% magnesium acetate in the cross-linking bath.

The obtained microscopy photographs are shown in Figure 3. The morphology of the particles in the cross-section is similar, revealing fibrillar formations that are obviously due to the polysaccharide.

Increasing the cross-linking agent amount determines a higher cross-linking degree predictably, as proven in photographs by increasing the density of the polymer matrix (Figure 3b), which still keeps the fibrillar character determined by gellan. The results are consistent with the variation of the swelling degree presented in the next section.

### 2.5. Swelling Behavior

**Figure 4:**
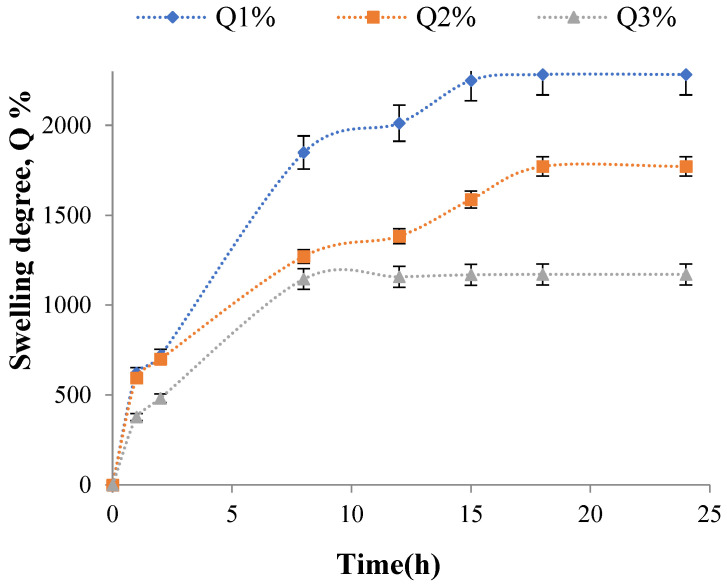
Variation of degree of swelling over time for three types of particles with immobilized α-amylase, obtained using different concentrations of magnesium acetate in the cross-linking bath (Q1%-A3, Q2%-A4, and Q3%-A5 magnesium acetate).

The gellan particles obtained by ionotropic cross-linking show an evident hydrogel character. Hence, their swelling characteristics in an aqueous environment are essential for their stability and further use in repeated hydrolytic processes. A hydrogel-type matrix allows, *a priori*, the substrate diffusion within the particles for contact with the immobilized enzyme and the diffusion of the hydrolysis products outside the polymer matrix. Figure 4 shows the variation of the swelling degree for samples with 3 mg of enzyme encapsulated obtained using different concentrations of Mg^2+^ in the cross-linking bath (A3, A4, and A5).

The degree of swelling value in the particles increases with the decrease in the cross-linking degree (the higher value was recorded for sample A3 with a magnesium acetate concentration in the cross-linking bath of 1%). In a previous work [92], in which we used ionically cross-linked gellan particles with different concentrations of magnesium acetate to immobilize yeast cells, the swelling degree was evaluated for the plain particles without the bioactive principle encapsulated. Previous results showed that the swelling degree values for particles cross-linked with 2 and 3% magnesium acetate were 1756 and 1631%, respectively, after approximately 8 h. For gellan particles containing encapsulated enzymes, it is observed that the swelling degree value after 24 h was 1772% for those cross-linked with 2% magnesium acetate and 1171% for those cross-linked with 3% magnesium acetate. The swelling degree values for gellan particles with encapsulated enzymes after 8 h were 1271% for those cross-linked with 2% magnesium acetate and 1146% for those cross-linked with 3% magnesium acetate. There is a possibility that the time required to perform the kinetics of the degree of swelling is not enough for the gellan particles cross-linked with Mg^2+^ ions without the bioactive principle immobilized to reach equilibrium. This hypothesis is also confirmed by the fact that for the same particle type but with encapsulated yeast cells, swelling degree kinetics occurred up to 36 h.

The encapsulated α-amylase influences the value of the swelling degree. The free amino groups within the enzyme can interact through electrostatic interactions with the carboxylic groups within the gellan, forming polyelectrolytic complexes. These polyelectrolyte complexes determine a slight increase in the cross-linking degree that can cause a slightly lower value of the swelling degree for samples with α-amylase immobilized. Additionally, the gellan particles cross-linked with Mg^2+^, in which the α-amylase was immobilized, were obtained using an acetate buffer solution (pH = 5.6; 0.05 M), which could obtain a stronger hydrogel compared to the hydrogels obtained at neutral pH, and the swelling degree value could also decrease. The research carried out by Kanyuck et al. [100] confirmed the above hypothesis. They showed that the swelling degree value was lower for gellan rich in acyl groups (high acyl gellan) when a concentration of 50 mM KCl was used in the swelling medium compared with the swelling degree value obtained for the same hydrogel in water [100].

In general, an increase in the ionic strength by using monovalent cations or by adding acidic solutions to the medium reduces the repulsion between the helices in gellan due to the reduction in the formation of carboxylate groups (COO^−^), and it causes a stronger interaction between the helices in the gellan structure. Consequently, hydrogels formed at neutral pH are more fragile and deformable than hydrogels obtained at acidic pH or with solutions containing monovalent ions [78,101,102].

### 2.6. Determination of the Michaelis–Menten Constant, the Maximum Rate of Hydrolysis V_max_, and the Constant K_m_

**Figure 5:**
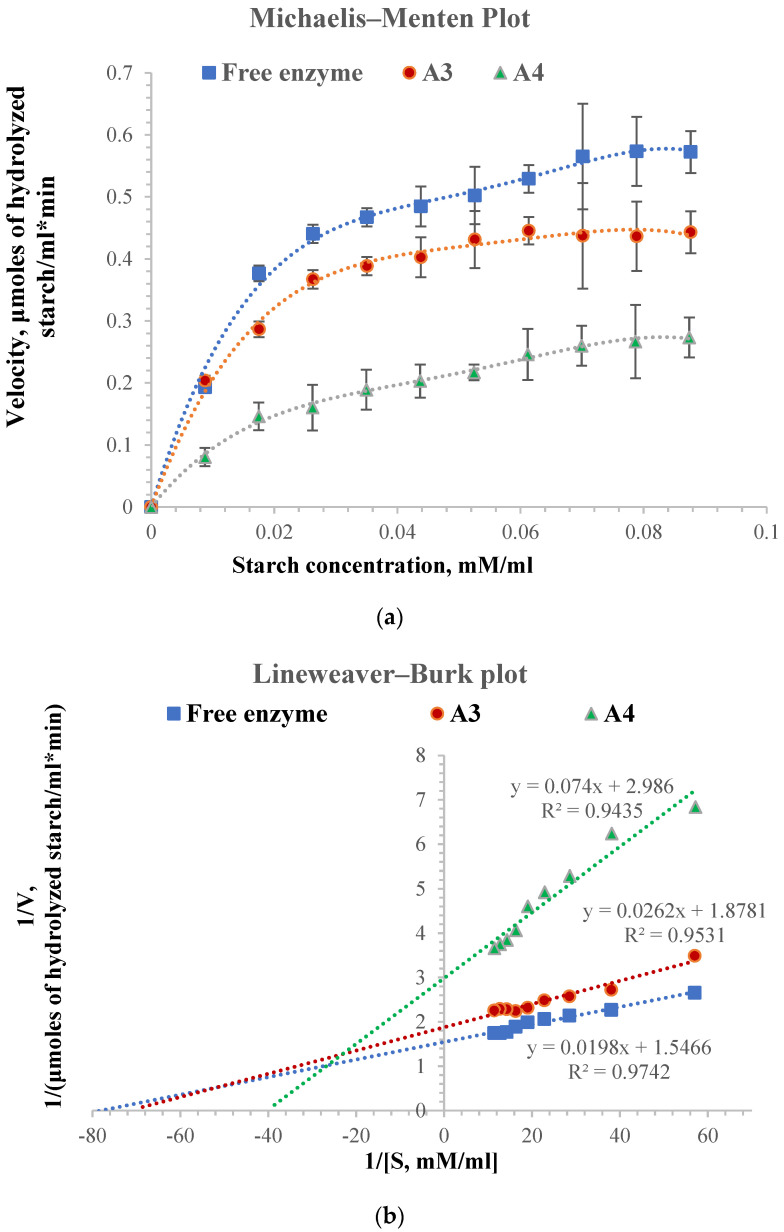
Michaelis–Menten kinetics (**a**) and Lineweaver–Burk plot (**b**) for free enzyme and enzyme immobilized in magnesium acetate cross-linked gellan particles A3 and A4.

The general principles of the kinetics of chemical reactions also apply to reactions catalyzed by enzymes, but they also have a characteristic of their own, namely substrate saturation. The Michaelis–Menten equation expresses the mathematical relationship between the initial rate of an enzyme-catalyzed reaction, the substrate concentration, and specific enzyme characteristics. Figure 5 shows the Michaelis–Menten kinetics for free and immobilized α-amylase in gellan particles cross-linked with different magnesium acetate concentrations (1% and 2%, respectively) and the Lineweaver–Burk plot. The values of the K_m_ constant and the maximum velocity, V_max_, for the analyzed samples are shown in Table 2. The same amount of free and immobilized enzyme of 0.16 mg was used to perform the Michaelis–Menten kinetics.

The K_m_ constant’s value shows the enzyme’s affinity for the substrate. A high value of the K_m_ constant suggests a lower affinity of the enzyme for the substrate. In general, the maximum velocity value is higher for the free enzyme than for the immobilized one, and the K_m_ constant is lower for the free enzyme than for the immobilized one [61,103]. As shown in Figure 5, the hydrolysis velocity of the immobilized enzyme is lower than that of the free enzyme, probably caused by both small perturbations in the enzyme structure by immobilization and the cross-linking degree of the particles, which leads to the slower diffusion of the substrate inside the particles [92,104].

From the Michaelis–Menten kinetics, the V_max_ (maximum velocity) for free enzyme and the apparent V_max_ for immobilized enzyme were determined. Its value for the free enzyme was 0.65 µmoles hydrolyzed starch/mL·min, and it can be seen from Table 2 that it decreases for the immobilized enzyme to a value of 0.53 µmoles hydrolyzed starch/mL·min for the A3 sample and 0.33 hydrolyzed starch/mL·min for A4 sample. The value of the constant K_m_ or K_m_′ increases with the decrease in starch hydrolysis velocity from 0.0128 mM starch/mL for the free enzyme to 0.0139 mM starch/mL for the enzyme immobilized in A3 particles and 0.0248 mM starch/mL for the enzyme immobilized in A4 particles. The higher affinity for the substrate of the enzyme immobilized in A3 particles compared to the affinity of the enzyme immobilized in A4 particles can be explained by a more enhanced diffusion of the starch solution within the hydrogel particles. The A3 sample containing 1% magnesium acetate in the cross-linking bath has a lower cross-linking degree than the A4 sample, which contains 2% magnesium acetate. The results are consistent with the swelling degree values, which depend on the diffusion of the substrate within the particles to the enzyme molecules.

In the case of the immobilized enzyme, the literature mentions a decrease in the affinity for the substrate (starch), which can be caused by the enzyme structural changes that occur during immobilization, the appearance of steric hindrance phenomena due to the support used, and the effects caused by a slower diffusion [105]. The α-amylase immobilized in sodium alginate particles also shows lower activity than the free enzyme. Talekar S and Chavare S found the value for apparent V_max_ and K_m_ to be 0.93 mg/mL and 2.30 μmole/min for the free enzyme and 1.12 mg/mL and 1.83 μmole/min for immobilized enzyme. They were used for the immobilization of α-amylase (diastase), and after immobilization, the substrate affinity of α-amylase decreased, which might be due to the lower accessibility of the substrate to the active site of the immobilized enzyme [103].

For the enzyme immobilized in A3 particles, the apparent K_m_’ value is close to the value of the free enzyme, meaning that their affinities are also close. The results show that immobilization did not modify the enzyme’s tertiary structure, and its affinity for the substrate was not substantially changed. However, it depends on the particles’ cross-linking degree and, therefore, on the diffusion of the substrate inside the particles.

### 2.7. Influence of pH and Temperature on Free and Immobilized Enzyme Activity

**Figure 6:**
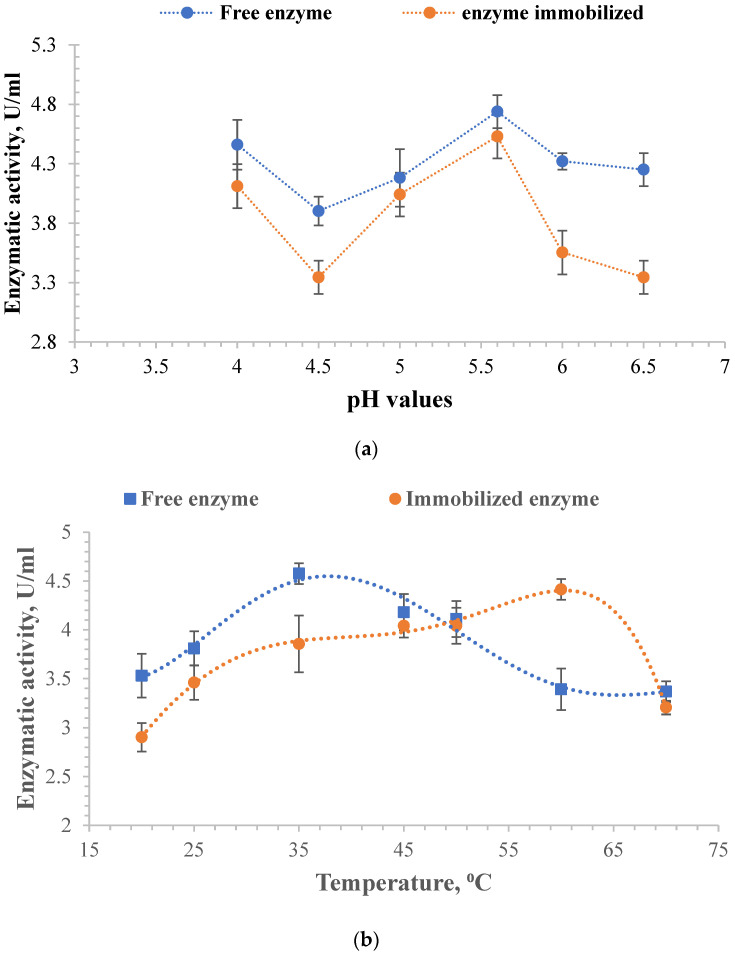
Influence of pH (**a**) and temperature (**b**) on enzyme activity for free and immobilized enzyme.

The determinations were made to establish the optimal value of the two parameters, which ensures maximum enzyme activity. The amount of free α-amylase was the same as that immobilized in A3 particles for all tests. Figure 6 shows the influence of pH and temperature on enzymatic activity.

The free enzyme has higher enzymatic activity than the immobilized one, as was found from the evaluation of the constant K_m_ and the maximum velocity V_max_. However, in both cases, it is observed that the maximum value of the enzymatic activity was reached at pH = 5.6 (Figure 6a). The optimum α-amylase activity was found to be in the pH range of 4.5 to 7. Decreasing the pH of the enzyme solution below this range results in a decrease in enzyme activity. Above pH 7, α-amylase activity rapidly decreases because the amino groups are no longer protonated or their concentration is too low [106].

By immobilization, the enzyme’s resistance to temperature increased up to the value of 60 °C. It can be observed from Figure 6b that the enzymatic activity of the immobilized enzyme increases slightly from 20 °C to 25 °C, then remains constant up to 50 °C, and increases again at 60 °C. Then, its activity suddenly decreases due to the possible degradation of the polymer matrix. Moreover, the free enzyme activity appears superior to the immobilized one except for its value at 60 °C, where the enzyme activity for the immobilized α-amylase is superior to the free one. The findings align with previously published research on immobilized α-amylase. For instance, Ahmed, N E et al. found that immobilized α-amylase remained stable at 60 °C for 20 min [107]. Similarly, El-Banna, T E demonstrated the thermal stability of immobilized α-amylase at 60 °C for 10 min [108]. Additionally, Hemanchi, H K and Sanjay N P found that exposing the beads to 60 °C for 60 min resulted in 40% relative activity for immobilized α-amylase [109]. The temperature at which the free enzyme had maximum enzymatic activity was 35 °C. At temperatures higher than 35 °C, there is a reduction in catalytic activity, indicating changes in the enzyme’s conformational structure, causing the denaturation of the free enzyme, which is reversible at temperatures up to 60 °C. Several irreversible reactions can also occur at higher temperatures, including deamidation, peptide bond cleavage, and denaturation, leading to enzymatic degradation [110,111]. The enzyme’s tertiary structure is altered at higher temperatures, leading to its slow degradation [92,112].

### 2.8. The Influence of NaCl Concentration on the Degree of Inhibition of Enzyme Activity for Free and Immobilized α-Amylase

As a function of the concentration, sodium chloride (NaCl) can affect the enzymatic activity of α-amylase. In humans, NaCl is found to be about 0.4% by weight, and the salt concentration in the blood is about 0.9%. At low concentrations, sodium chloride does not affect amylase activity. However, at higher concentrations (2 M and 5 M), it slows amylase activity because changes in salinity can disrupt the molecular structure of the enzyme, its intermolecular hydrogen bonds, solubility, binding, and stability, which lead to the denaturation of the enzyme [113,114].

In order to evaluate the behavior of free and immobilized α-amylase in the presence of different concentrations of salt, solutions with different concentrations of NaCl between 0.5 M and 4 M were used in which the free and immobilized α-amylase were incubated for 2 and 24 h, respectively, at 4 °C. The enzyme was immobilized in the A4 particles (Table 1). The amount of immobilized and free enzyme used was the same (0.25 mg) for each concentration of NaCl used to perform this test.

**Figure 7:**
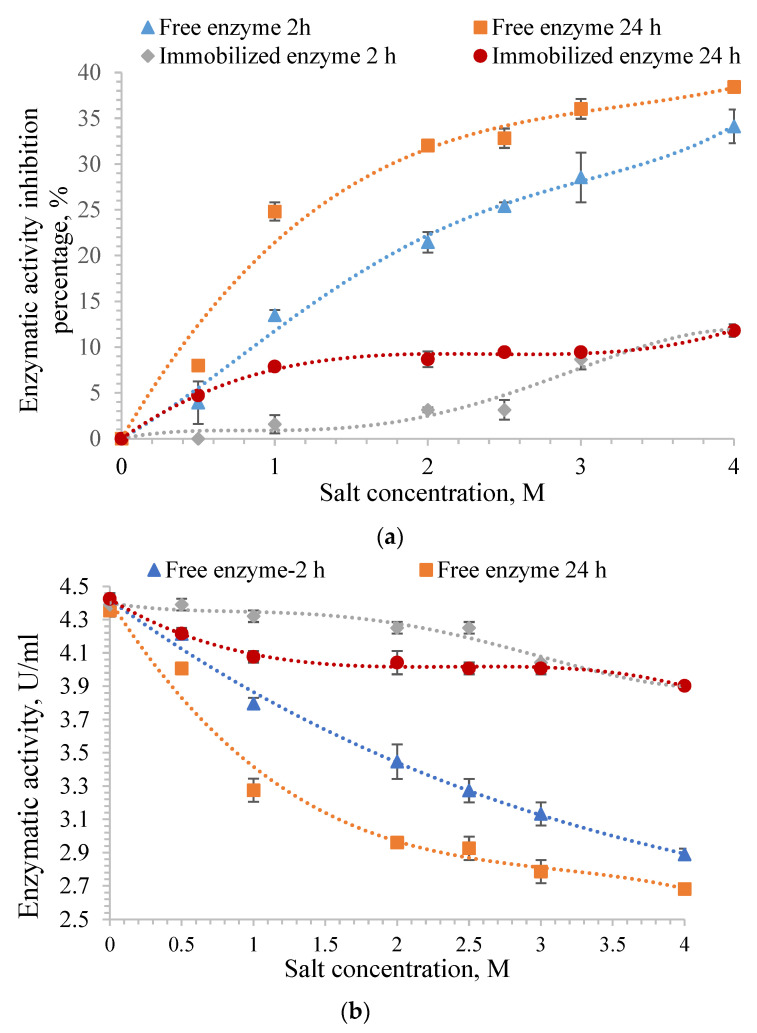
The α-amylase inhibition percentage on NaCl for free and immobilized α-amylase (**a**) and enzyme activity in the presence of different salt concentrations (**b**).

α-amylase shows maximum activity in the absence of NaCl. Figure 7 shows the obtained results. It can be observed from Figure 7a that the inhibition percentage of the enzyme activity for both free and immobilized amylase after incubation in saline solutions increases with increasing NaCl concentration. Figure 7b shows the enzyme activity that decreases with increasing salt concentration.

Regarding the results obtained for the free enzyme, it was observed that the enzymatic activity inhibition percentage in the same salt concentration, 4 M, was 34% for the enzyme incubated for 2 h and 38% for the enzyme incubated for 24 h.

Figure 7 proves that the polymer matrix protects the immobilized enzyme, and its enzymatic activity is preserved; the inhibition percentage of α-amylase immobilized in the gellan particles is much lower than that of the free enzyme. The maximum inhibition percentage was 11% for the immobilized α-amylase regardless of the incubation period in the 4 M NaCl solution, and it was observed that the polymer matrix protected the enzyme. The inhibition percentage of enzymatic activity for free enzyme incubated in different NaCl solution concentrations was higher when both the incubation time and the ionic strength of the incubation medium increased. It was observed that the enzymatic activity inhibition percentage of free α-amylase increased by 100% after 24 h of incubation in a 0.5 M NaCl solution when compared with the inhibition percentage obtained after 2 h of incubation in the same solution. It could be noted that high salt concentrations require less time to inhibit the enzymatic activity of α-amylase.

The results are comparable to those from the literature [114] and are determined by the enzyme’s denaturation in the presence of high salt concentrations. The inhibition percentage of enzymatic activity for the immobilized α-amylase increases after 2 h or 24 h with increasing NaCl concentration, but it is observed to have a much lower value than the free enzyme.

The enzymatic activity of the immobilized α-amylase was not affected when the enzyme was incubated for 2 h in a 0.5 M NaCl solution. It decreased slightly after 24 h of incubation due to the diffusion of a small amount of salt solution within the hydrogel particles. However, they remain at high values compared to those obtained for the free enzyme because the gellan particles protect α-amylase, and its tertiary structure is not affected.

### 2.9. Enzymatic Activity Assay of the Immobilized Enzyme in Several Hydrolytic Cycles

**Figure 8:**
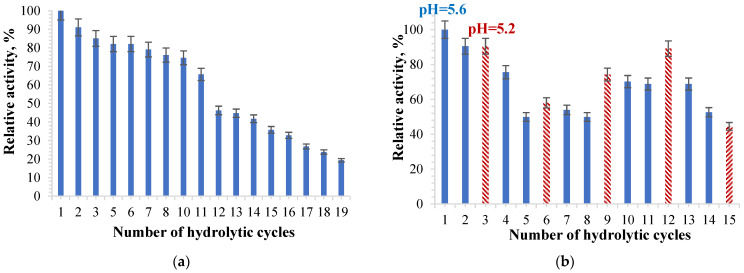
Relative activity determinations in repeated hydrolytic cycles for particles with immobilized α-amylase (3 mg/mL), using starch as substrate at (**a**) pH 5.6 and (**b**) pH 5.2 and pH = 5.6. The temperature at which the catalytic activity of the particles was tested was 35 °C. The duration between hydrolytic cycles was 10 min.

Particles with immobilized α-amylase were tested in several repeated hydrolysis cycles in order to check if they could be reused without essential activity loss. The sample selected for determination was A4, with the ionic cross-linking being carried out with a 2% magnesium acetate concentration solution. Figure 8 shows the results obtained after repeated hydrolysis with α-amylase immobilized in hydrogel particles based on gellan.

Figure 8a shows that the α-amylase immobilized in the gellan particles retains its activity after several hydrolytic cycles; it does not suffer a significant decrease until the 11th cycle, after which it starts to decrease. After each hydrolysis cycle, the particles were separated by filtration, washed with acetate buffer solution: pH = 5.6, and kept in this acetate buffer solution for 5 min until the next hydrolysis cycle.

Talekar and Chavare [61] used α-amylase immobilized in alginate particles ionically cross-linked with calcium ions in 10 repeated hydrolysis cycles, and the α-amylase activity gradually decreased from one cycle to the next.

Based on the obtained results, a more significant number of hydrolytic cycles can be obtained using immobilized α-amylase in hydrogel particles based on gellan. The gellan matrix is more resistant than the alginate matrix. Additionally, in our research, the Mg^2+^ was used for cross-linking and not Ca^2+^, which gives the hydrogel particles a stronger and more stable structure than the one based on alginate. Previous research that focused on the influence of metal ions on the activity of α-amylase showed that the enzymatic activity is maximum in the presence of magnesium ions [115]. The researchers showed that the enzyme immobilized in alginate particles cross-linked with CaCl_2_ retains only 35% of the original enzyme activity after 10 hydrolytic cycles [103]. In the case of the enzyme immobilized in gellan particles ionically cross-linked with Mg^2+^ ions, the enzyme retains 75% of the initial activity of the immobilized enzyme after 10 hydrolytic cycles. α-amylase immobilized in an amidrazone acrylic fabric was reused over 15 hydrolytic cycles, and 53% of the enzyme activity was maintained [43]. In the case of α-amylase encapsulated in ionically cross-linked gellan particles, after 15 hydrolysis cycles, the relative activity of the enzyme decreases to approximately 28% (Figure 8a). For the same samples treated with acetate buffer solution: pH = 5.2, 0.1 M, the relative activity of α-amylase decreased to 38% after 15 cycles of hydrolysis (Figure 8b). The researchers identified two major factors for the immobilized enzyme to lose its catalytic activity, namely the change in the stereochemical structure of the enzyme and the diffusion of the enzyme during the recycling process [43]. Frequent interaction of the substrate with the active site of the immobilized enzyme causes distortion in the active site, causing a loss of activity over time [116,117].

In order to establish whether the enzymatic activity value could increase close to the initial value after testing the α-amylase-loaded gellan hydrogel particles from one hydrolysis cycle to another, a test in which the immobilized α -amylase was treated with a more acidic solution (pH = 5.2) after two hydrolysis cycles that took place at pH = 5.6 was performed. Thus, after two hydrolysis cycles using the acetate buffer solution at pH 5.6, the samples with immobilized enzyme were washed with the acetate buffer solution at pH = 5.2 and maintained at this pH for 30 min. The obtained results are presented in Figure 8b. It can be observed that enzyme activity increases after each hydrolysis cycle catalyzed by the particles that were kept in the acetate buffer solution at a pH = 5.2. The number of protonated amino groups in the enzyme can increase by immersing the gellan particles with immobilized α-amylase in a slightly more acidic solution. Thus, intermolecular bonds, such as the electrostatic interactions of the amino groups of the enzyme with free carboxylic groups within the gellan-based polymeric matrix, could occur, which determines the obtainment of a more stable structure in the particle hydrogel.

Moreover, the slight increase in enzymatic activity of α-amylase immobilized in gellan particles after treatment with a slightly more acidic solution may cause higher stability regarding the hydrogel particles that no longer tend to disintegrate. Furthermore, α-amylase remains immobilized in gellan particles and does not diffuse into the hydrolysis medium. Figure 8b shows that the enzymatic activity of the particles with immobilized α-amylase is higher than that in which the particles with immobilized α-amylase were not treated with the solution at pH = 5.2 (Figure 8a).

## 3. Materials and Methods

### 3.1. Materials

Gellan (M_w_ = 2–3 × 10^5^ Da) was purchased from Kelkogel; α-amylase from porcine pancreas with a molecular weight of 55 kDa, magnesium acetate, soluble starch, bovine serum albumin, and Folin-Ciocalteu’s phenol reagent were purchased from Sigma Aldrich; acetic acid, hydrochloric acid, sodium acetate, sodium carbonate, sodium hydroxide, copper sulfate (CuSO_4_·5H_2_O), Na and K double tartrate solution, and iodine solution were purchased from the Chemical Company, Romania.

### 3.2. Methods

#### 3.2.1. Free Enzyme Tests

- Preparation of Free Enzyme Solution

A stock solution of α-amylase with a 0.3922 mg/mL concentration was prepared by dissolving the enzyme in 0.1 M acetate buffer solution at pH = 5.6. The prepared stock solution was stored in the refrigerator at 4 °C for use in future analyses.

- Determination of the Enzymatic Activity of Free α-Amylase

The enzymatic activity of free and immobilized α-amylase was determined using the modified colorimetric method with starch and iodine solution [88,118]. The amount of unhydrolyzed starch that is the basis of the evaluation of the enzymatic activity is established based on the calibration curves shown in Figure S3, which have the equations y = 0.1508x (calibration curve drawn for low starch concentrations). This calibration curve was used for the enzyme tests where low concentrations of starch were used (the total concentration used in the enzyme tests being 0.57 mg/mL in the hydrolysis medium of 7 mL). The calibration curve with the equation y = 0.0791x (calibration curve plotted for high starch concentrations) was used to determine the constants K_max_ and V_max_ from the Michaelis–Menten equation because the starch concentrations used in this case were higher compared with the hydrolysis medium used for enzymatic activity determination (the maximum concentration used was 4.29 mg/mL in the hydrolysis medium of 7 mL). Schematically, Figure S4 shows the working method for enzymatic activity determination.

The working method for each enzyme activity determination was as follows:

1 mL of starch solution was added over 6 mL of 0.1 M buffer solution (generally acetate buffer solution, pH = 5.6), after which 200 µL of α-amylase of different concentrations was added. The enzyme reaction will be optimized according to temperature, pH, and hydrolysis time by measuring the enzymatic activity. A 200 μL sample is taken from the reaction mixture and added to a test tube containing 1 mL of 0.5 N HCl used to stop the hydrolysis reaction. The total starch concentration in the 7 mL solution was 0.57 mg/mL. The determination was carried out via colorimetrics (starch in the presence of iodine turns blue-violet). The enzymatic activity was tested, as described above (Figure S4), and the absorbance was read after 20 min at a wavelength of 620 nm using a UV-VIS spectrophotometer type BOECO S-22. Enzymatic activity expressed in μmoles hydrolyzed starch/min·mL or U/mL was determined, and it is described using the relationship below:

Enzyme Activity (μmol hydrolyzed starch/min·mL) or (U/mL) = [Hydrolyzed starch (µmoles) − Concentration of starch that remains after hydrolysis (µmoles)] × Total Reaction Volume/(Reaction time (min)) × (Enzyme volume (mL))(1)

The amount of hydrolyzed starch (mg) was determined by the difference between the total amount of starch (mg) initially found in the reaction and the quantity of starch determined after the hydrolysis reaction from the calibration curve (mg) (Figure S3a). The micromoles of hydrolyzed starch were determined by the report between the amount of hydrolyzed (mg) starch divided by the structural unit molecular weight of starch and then multiplied by 1000.

- Establishing the Time Required for an Enzymatic Reaction

Free enzyme assays were performed to determine enzyme activity as a function of starch concentration. A total of 1 mL of the starch stock solution (with a concentration of 0.4%) was added over 6 mL of acetate buffer solution: 0.1 M, pH = 5.6, in a beaker under stirring. A volume of 200 μL of the enzyme stock solution was taken from the stock solution (0.3922 mg/mL) and added to the previous mixture. A 200 µL sample was taken each minute until 14 min. It was added to a test tube containing 1 mL of HCl 0.5 N used to stop the hydrolysis reaction, and, as described in Figure S4, 200 µL of this mixture was added to a test tube containing 5 mL of 0.05 N iodine solution. The reaction temperature was 35 °C. The amount of non-hydrolyzed starch was determined spectrophotometrically at 620 nm, from which the amount of hydrolyzed starch was determined. Enzymatic activity was determined, as described above, using Equation (1). The reaction rate represents the amount of starch converted into µmoles of hydrolyzed starch per minute.

- Enzyme Activity Calibration Curve

A calibration curve for the enzyme is required to show the initial rate (µmoles of hydrolyzed starch/minute) using several enzyme concentrations and a constant starch concentration of 0.4% (*w*/*v*). The working method was as follows: 1 mL of starch solution was added over 6 mL of acetate buffer solution: pH = 5.6. Several solutions with different enzyme concentrations were prepared. A total of 200 μL of each α-amylase solution of different concentrations was added over the mixture containing the starch solution (the total volume was 7.2 mL). The enzyme concentrations used were 0.03922 mg/mL, 0.05971 mg/mL, 0.08019 mg/mL, 0.1399 mg/mL, 0.1966 mg/mL, 0.2495 mg/mL, 0.2994 mg/mL, 0.3458 mg/mL, and 0.3922 mg/mL. A total of 200 μL of each enzyme concentration was left in contact with the starch solution for 10 min at 35 °C. The enzymatic activity and hydrolysis reaction rate for each enzyme concentration were determined.

#### 3.2.2. Preparation of Particles Containing α-Amylase Immobilized in Gellan Matrix

**Figure 9:**
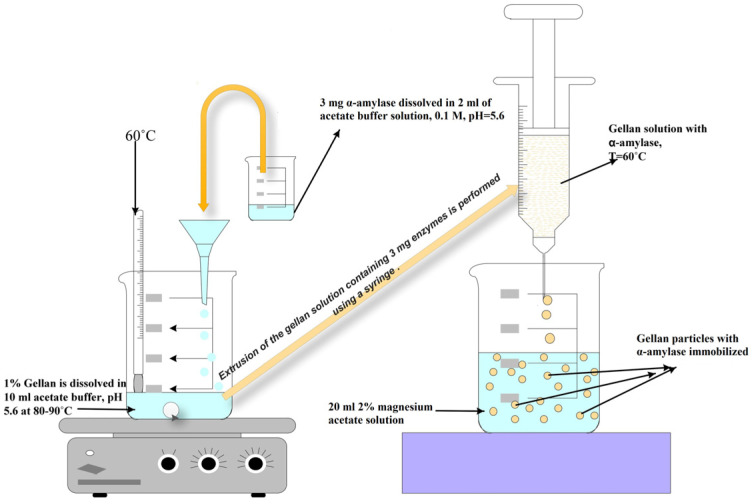
Schematic presentation of the preparation of gellan particles with 3 mg of immobilized α-amylase.

Gellan particles with immobilized α-amylase were obtained by ionic cross-linking with magnesium acetate using the extrusion method. Schematically, the obtainment process is shown in Figure 9. Several concentrations of α-amylase were used to determine the optimal concentration that can be immobilized in gellan particles. A total of 0.1 g of gellan (1%) was dissolved at 85 °C, under stirring, in 10 mL of acetate buffer solution at a pH of 5.6 (0.05 M). Thus, the solution was cooled to a temperature of 60 °C when the amount of α-amylase required for immobilization dissolved in acetate buffer solution, pH = 5.6, was added under stirring. The ionotropic cross-linking bath contained 20 mL of different magnesium acetate solution concentrations (1%, 2%, and 3%). The polymer solution containing enzyme was extruded at 60 °C dropwise using a syringe and a needle (23 gauge) in the cross-linking bath. At a lower temperature, the gellan solution thus obtained becomes very viscous, and the enzymatic activity of α-amylase decreases (Table S1—supplementary information). The extruded droplets were instantly transformed into spherical, swollen hydrogel particles upon contact with the cross-linking agent solution. The particles with immobilized α-amylase were kept in the refrigerator at 4–6 °C until the subsequent tests were performed. Schematically, Figure 9 shows the preparation process of immobilized α-amylase using the concentration of enzymes and the optimal temperature for obtaining them.

#### 3.2.3. Determination of the Optimal Temperature and Amount of α-Amylase That Can Be Encapsulated in Gellan Particles by Determining the Immobilization Efficiency and by Testing the Enzyme Activity

- Determination of Immobilization Efficiency, E_f_%

The immobilization efficiency, E_f_%, of α-amylase was determined based on the amount of enzyme in the supernatant. The Lowry method was used to determine the proteins in the supernatant. Figure S5 shows the calibration curve of bovine serum albumin used as a standard, which is based on the amount of α-amylase that was not immobilized, was determined. In the Lowry method, the blue color results from the complexation of the peptide bond with copper sulfate and the reduction in the Folin-Ciocalteu reagent by the tyrosine and tryptophan residues in the protein [119]. The calibration curve of albumin had the equation: y = 0.0007x, R^2^ = 0.9988, and the immobilization efficiency of the samples was determined using Equation (2):

(2) $$E_{i}=\frac{m_{Ei}-m_{Es}}{m_{Ei}}\times 100$$

where the m_Ei_ is the amount of initial enzyme, m_Es_ is the amount of enzyme found in the supernatant after extrusion.

- Determination of the Optimal Amount of Enzymes that Can Be Immobilized

In order to determine the optimal enzyme amount that can be immobilized in the gellan particles, several concentrations of α-amylase were used (1, 2, 3, 3.5, 4, and 5 mg/mL, respectively). The total volume of solution added to the gellan solution containing α-amylase was 2 mL. The amount of protein in the supernatant was determined for each sample. The immobilization efficiency was calculated based on Equation (2), and based on the obtained values, the optimal enzyme concentration that can be immobilized was determined. Enzymatic activity was tested using one gram of swollen particles with different enzyme concentrations immobilized for each determination, and Equation (1) was used to calculate it. The results obtained can be found in Table 1. All the determinations were performed in triplicate. The results are given as average values ± Stdev.

- Determination of the Optimal Temperature for the Immobilization of α-Amylase

In order to determine the optimal immobilization temperature, type A3 particles were used (Table 1). The enzyme solution was added at different temperatures (40 °C, 50 °C, and 60 °C) to the gellan solution before extrusion in 1% magnesium acetate solution. Enzymatic activity testing of the particles with α-amylase immobilized was carried out by adding one gram of swollen hydrogel particles to the substrate prepared from 6 mL acetate buffer solution: pH = 5.6 and 1 mL starch 0.4%. The particles were maintained in contact with the starch solution for 10 min, and the enzymatic activity was tested at 35 °C. The working method for testing the enzymatic activity is presented schematically in Figure S4. The enzymatic activity was calculated using Equation (1), and the results can be found in Table S1. All the determinations were performed in triplicate. The results are given as average values ± Stdev.

#### 3.2.4. FT-IR Spectroscopy

FT-IR spectra were recorded using a Shimadzu IR Affinity 1S spectrometer according to the KBr method for gellan, α-amylase, and the particles with α-amylase immobilized. Potassium bromide tablets were obtained by mortaring the samples in powder form (10 mg) with anhydrous KBr; the resulting mixture was pressed using a hydraulic press. For each sample, several scans were performed in the range of 4000–400 cm^−1^, with a resolution of 4 cm^−1^. The highlighted bands in the absorption spectrum were assigned to the corresponding functional groups present in the analyzed sample. The spectra were recorded for gellan, α-amylase, and sample A4.

#### 3.2.5. Particle Morphology (Scanning Electron Microscopy)

Particles containing α-amylase were characterized by scanning electron microscopy to determine their morphology. The particles were dried, cut in cross-sections, metalized with gold using a sputter deposition device, and analyzed using a Vega Tescan instrument.

#### 3.2.6. Swelling Degree

The obtained particles have a hydrogel character, so it was considered useful to determine their ability to absorb water, which is usually quantified using the swelling degree (Q%).

Q% was determined gravimetrically, as described in [86], with a few modifications for both types of particles (with and without immobilized α-amylase). First, the samples were dried until they had constant weight. A certain weighed number of particles (M_dry_) was immersed in 5 mL of bi-distilled water, which was removed by filtration at 1 h intervals, and the water excess from the surface of the swollen particles was absorbed with filter paper. The water retained by the particles (M_water_) is the difference between the weight of the swollen particles (M_swollen particles_) and the weight of the dry particles (M_ary_). After weighing, the samples were reintroduced into the swelling medium, repeating the operation every hour until equilibrium was reached. The swelling degree was expressed as the ratio between the amount of water existing in the particles at each time interval and the amount of completely dry particles (Equation (3)):

(3) $$Q\%=\frac{M_{water}}{M_{dry}}\times 100$$

The swelling degree was determined in triplicate. The standard deviation is within ± 5%.

#### 3.2.7. Determination of the Michaelis–Menten Constant and the Maximum Hydrolysis Rate of Free and Immobilized α-Amylase

The Michaelis–Menten theory assumes that enzyme E combines with substrate S to form the enzyme-substrate ES complex. This complex decomposes later, obtaining the free enzyme (E) and the product (P). The Michaelis–Menten equation expresses the mathematical relationship between the initial speed of an enzyme-catalyzed reaction, the concentration of the substrate, and the specific characteristics of the enzyme, being valid only for enzyme-catalyzed reactions with a single substrate (Equation (5)), where v_0_ is the initial reaction rate; V_max_ is the maximum reaction rate; K_m_ is the Michaelis–Menten constant; [S] is the substrate concentration.

(4) $$E+S\begin{array}{c} \overset{k_{1}}{\to }\\ \underset{k_{-1}}{\leftarrow } \end{array}\mathrm{ES}\overset{k_{2}}{\to }E+P$$

where E is the enzyme concentration, S is the substrate concentration, *k*_1_ is the reaction rate constant between the enzymes and substrate for the formation of the ES complex, *k*_−1_ is the reaction rate constant for the reverse reaction, and *k*_2_ is the reaction rate constant for dissociation of the complex ES. The rate of reactant consumption or product formation can be expressed as [120]:

(5) $$v_{0}=\frac{V_{\max}\times [S]}{K_{m}+[S]}$$

Several starch concentrations between 3 and 30 mg/mL were used to determine the K_m_ constant and V_max_ maximum reaction velocity. For the determination of the Michaelis–Menten kinetics, the procedure was as follows: several starch solutions of different concentrations ranging from 3 mg/mL to 30 mg/mL were prepared, which were converted into mM substrate/mL knowing that the molecular weight of the structural unit from soluble starch. A total of 1 mL of each starch solution was added to 6 mL of acetate buffer solution, pH = 5.6, at a temperature of 35 °C. The amount of immobilized enzyme and the amount of free enzyme used in the hydrolysis reaction was the same (the amount of free enzyme added was 0.16 mg in a volume of 400 μL from enzyme stock solution, and the amount of immobilized enzyme in 1 g of particles was also 0.16 mg enzyme). The rate of hydrolysis of starch (µmoles hydrolyzed starch/minute) depends on the concentration of starch used. It was determined for the free enzyme (the stock solution had a concentration of 39.22 mg/mL) and for the immobilized α-amylase in particles. The temperature at which the determinations were made was 35 °C. The reaction rate was determined after 10 min and expressed as hydrolyzed starch/minute. A straight line (1/S vs. 1/V) was drawn based on the obtained results. The slope was equal to $\frac{-\left[S_{0}\right]}{K_{m}}$, and the intercept was equal to $\frac{V_{max}}{K_{m}}$, from where the kinetic parameters for the free and immobilized enzyme V_max_ and K_m_ were calculated. The results obtained are shown in Table 2 and Figure 5. All the determinations were performed in triplicate. The results are given as average values ± Stdev.

#### 3.2.8. Influence of pH and Temperature on Enzymatic Activity

In order to determine the optimal pH at which the enzyme activity is maximum, different pH values were used in the hydrolysis bath, namely: 4, 4.5, 5, 5.6, 6, and 6.5. The temperature at which the starch hydrolysis was carried out was 35 °C, and for the immobilized α-amylase, the starch hydrolysis took place at 60 °C. In order to determine the maximum enzyme activity temperature, the enzymatic hydrolysis of starch was performed in the presence of the free enzyme or the immobilized enzyme using several temperatures between 20 and 80 °C. The pH used in the hydrolysis medium was 5.6. The free enzyme solution used had a concentration of 39.22 mg/100 mL (0.078 mg enzymes/0.2 mL). The initial amount of immobilized enzymes was 3 mg/mL, and 0.5 g of particles was used containing the same amount of α-amylase (0.078 mg) as in the case of the free enzyme. The procedure by which enzymatic activity was determined at different values of the two parameters is the one previously described (Figure S4, Equation (1)). All the determinations were performed in triplicate. The results are given as average values ± Stdev.

#### 3.2.9. The Influence of NaCl Concentration on the Degree of Inhibition of Enzyme Activity for Free and Immobilized α-Amylase

In order to determine the influence of the salt concentration on the degree of inhibition of the enzymatic activity for free α-amylase, a stock solution was prepared in 0.1 M acetate buffer solution: pH = 5.6 of 0.39g α-amylase/100mL. From this solution, 8.0 mL of α-amylase solution (containing 31.25 mg of enzymes) was taken and added to 25 mL volumetric flasks, over which 17 mL of NaCl solution with different amounts of NaCl was added to obtain the final concentrations of 0.5 M, 1 M, 2 M, 2.5 M, 3 M, and 4 M in a volume of 25 mL solution. The control solution containing 31.25 mg of enzymes in 25 mL of 0.1 M acetate buffer solution, pH = 5.6, was also prepared. The enzyme was incubated in the prepared solutions for 2 h (7 samples) and 24 h (7 samples) at a temperature of 4 °C before determining the enzymatic activity at 35 °C and its inhibition percentage. A volume of 200 μL of each prepared solution containing 250 µg of free enzyme is required for enzyme activity determination.

In order to determine the influence of salt concentration on the enzyme activity inhibition degree for immobilized α-amylase, 0.79g of A4 particles containing 250 µg of α-amylase was used. The particles were immersed in the previously mentioned salt solutions of different concentrations (25 mL) and were incubated for 2 h (7 samples) and 24 h (7 samples) before the enzyme activity assay at 60 °C. The inhibition percentage of enzymatic activity was determined as follows:

(6) Salt inhbition%=(AC−AP)AC×100

where

- *A_C_*: enzymatic activity assay of NaCl-free α-amylase solution/particles used as a control solution;
- *A_P_*: enzymatic activity for the samples used in which the free or immobilized enzyme was immersed in different concentrations of NaCl.

Determinations were performed in triplicate and are expressed as mean values ± stdev.

#### 3.2.10. Enzymatic Activity Testing of Particles Containing Immobilized Enzyme in Several Hydrolytic Cycles

A total of 1 mL of 0.4% starch stock solution in 6 mL of 0.1M acetate buffer solution at pH = 5.6 and 0.5 g of particles containing α-amylase (0.16 mg of enzyme) were used, and the temperature at which the enzymatic activity was determined (through the procedure previously described) was 35 °C. This temperature was chosen because the particles become unstable at 60 °C after the 4th cycle of hydrolysis. Each hydrolysis cycle lasts 10 min. The particles with immobilized α-amylase were tested over several repeated hydrolysis cycles. After each hydrolysis cycle, the particles were washed with 0.1 M acetate buffer solution, pH = 5.6, and kept in this solution for 5 min before reusing them in a new hydrolysis cycle. Acetate buffer solution: 0.1 M, pH = 5.2, was used to determine if the immobilized α-amylase could be reactivated. Thus, after every two hydrolysis cycles performed, the particles with immobilized enzyme were washed with 0.1 M acetate buffer solution at pH = 5.2 and maintained in this solution for 30 min until the next hydrolysis cycle. The results were expressed as relative activity % and were calculated by taking the enzyme activity of the first cycle as 100%. All the determinations were performed in triplicate. The standard deviation is within ±5%.

## 4. Conclusions

Stable gellan-based hydrogel particles containing immobilized α-amylase were obtained by ionotropic gelation using Mg^2+^ ions. The immobilization efficiency and enzymatic activity of the particles depend on the amount of enzyme used and the degree of cross-linking. The FT-IR spectra of the particles confirm that the enzyme was incorporated into the ionically cross-linked gellan particles. The free amino groups from the enzyme could interact electrostatically with the carboxylic groups within gellan, and the polyelectrolyte complexes were obtained. The swelling degree value in water decreases with increasing cross-linker concentration, resulting in higher cross-linking density. The morphology of the particles reveals fibrillar formations and the porosity of the matrix being reduced with the increase in the degree of cross-linking. The temperature and pH at which the immobilized enzyme activity is at maximum have the following values: T = 60 °C and pH = 5.6. In the case of the immobilized enzyme, the Michaelis–Menten constant shows a decrease in affinity for the support (starch). The enzyme’s affinity for the substrate also depends on the particle type used. It decreases for particles with a higher cross-linking degree due to the slow diffusion of the substrate to the enzyme molecules inside the particles. The immobilized α-amylase can be easily recovered from the hydrolysis medium, being able to be used in 11 repeated hydrolytic cycles without an essential decrease in enzyme activity (approximately 75% from the enzymatic activity was retained); this was gradually reduced until cycle 19. α-amylase immobilized in gellan particles can be regenerated via treatment in a more acidic environment. The results obtained are encouraging, as the prepared system can be an alternative to biotechnologies from the food or pharmaceutical industries.

## Figures and Tables

**Table 1:** Experimental plan for obtaining immobilized α-amylase *.

Sample Code *	α-Amylase Concentration, mg/mL	Magnesium Acetate Concentration, %	Immobilization Efficiency, E_f_%	Enzymatic Activity/g of Particles·mL
A1	1	2	66.7 ± 4.8	3.97 ± 0.3
A2	2	2	68.45 ± 6.6	4.36 ± 0.05
A3	3	1	63.49 ± 1.6	4.71 ± 0.05
A4	3	2	75.56 ± 5.1	4.28 ± 0.15
A5	3	3	89.68 ± 3.1	4.15 ± 0.05
A6	3.5	2	66.89 ± 0.6	3.83 ± 0.1
A7	4	2	66.07 ± 0.6	4.63 ± 0.05
A8	5	2	56.9 ± 1.7	3.66 ± 0.05

* Concentration of the gellan solution: 1%; pH of the acetate buffer solution used: 5.6; immobilization temperature: 60 °C.

**Table 2:** Values of the constant K_m_ and the maximum velocity V_max_ for free and immobilized enzyme.

Sample	K_m_ (mmoles)	V_max_ (µmoles Hydrolyzed Starch/mL·min)
Free enzyme	0.0128 ± 0.0028	0.65 ± 0.043
A3	0.014 ± 0.001	0.53 ± 0.011
A4	0.0248 ± 0.008	0.33 ± 0.03

## Data Availability

The data presented in this study are available upon request from the corresponding author.

## Supplementary Materials

The following supporting information can be downloaded at: https://www.mdpi.com/article/10.3390/molecules28124695/s1. Figure S1. Variation of starch hydrolysis rate over time using a constant concentration of free enzyme and starch. Figure S2. Variation of starch hydrolysis rate (expressed as µmoles of hydrolyzed starch/min) (a) and enzymatic activity (b) using different concentrations for free enzyme and a constant starch concentration. Table S1. Determination of the enzymatic activity for particles in which α-amylase was encapsulated at different temperatures; Figure S3. Calibration curves for starch using (a) concentrations less than 1 mg/mL and (b) concentrations up to 4 mg/mL. Figure S4. Schematic presentation of the working method for determining the activity of free or immobilized α-amylase; Figure S5. Bovine serum albumin (BSA) calibration curve.

## References

[1] Zhang H., Wang L., Shen Q., Wu B., Gao P. (2011). A novel approach for estimating the relationship between the kinetics and thermodynamics of glycoside hydrolases. Acta Biochim. Biophys. Sin..

[2] Chapman J., Ismail A.E., Dinu C.Z. (2018). Industrial Applications of Enzymes: Recent Advances, Techniques, and Outlooks. Catalysts.

[3] Karmee S.K. (2023). Moving towards the Application of Biocatalysis in Food Waste Biorefinery. Fermentation.

[4] Mohamad N.R., Marzuki N.H.C., Buang N.A., Huyop F., Wahab R.A. (2015). An overview of technologies for immobilization of enzymes and surface analysis techniques for immobilized enzymes. Biotechnol. Biotechnol. Equip..

[5] Reetz M.T. (2016). What are the Limitations of Enzymes in Synthetic Organic Chemistry?. Chem. Rec..

[6] Miłek J. (2021). Determination of Activation Energies and the Optimum Temperatures of Hydrolysis of Starch by α-Amylase from Porcine Pancreas. Molecules.

[7] Sky-Peck H.H., Thuvasethakul P. (1977). Human Pancreatic a-Amylase II. Effects of pH, Substrate and Ions on the Activity of the Enzyme. Ann. Clin. Lab. Sci..

[8] Bai Y., Atluri S., Zhang Z., Gidley M.J., Li E., Gilbert R.G. (2021). Structural reasons for inhibitory effects of pectin on α-amylase enzyme activity and in-vitro digestibility of starch. Food Hydrocoll..

[9] Tallapragada P., Dikshit R., Jadhav A., Sarah U. (2017). Partial purification and characterization of amylase enzyme under solid state fermentation from Monascus sanguineus. J. Genet. Eng. Biotechnol..

[10] Nijabat A., Manzoor S., Faiz S., Naveed N.H., Bolton A., Khan B.A., Ali A., Simon P. (2023). Variation in Seed Germination and Amylase Activity of Diverse Carrot (*Daucus carota* (L.)) Germplasm under Simulated Drought Stress. HortScience.

[11] Shohreh A., Behrouz Z., Seyedeh Fatemeh S.M., Kaveh K., Morteza M., Swapnoneel R., Ghasem H.S. (2021). Highly Efficient Computationally Derived Novel Metagenome α-Amylase With Robust Stability Under Extreme Denaturing Conditions. Front. Microbiol..

[12] Pan S., Yao T., Du L., Wei Y. (2020). Site-saturation mutagenesis at amino acid 329 of Klebsiella pneumoniae halophilic α-amylase affects enzymatic properties. J. Biosci. Bioeng..

[13] Che Hussian C.H.A., Leong W.Y. (2023). Thermostable enzyme research advances: A bibliometric analysis. J. Genet. Eng. Biotechnol..

[14] Mesbah Noha M. (2022). Industrial Biotechnology Based on Enzymes From Extreme Environments. Front. Bioeng. Biotechnol..

[15] Guzik U., Hupert-Kocurek K., Wojcieszyńska D. (2014). Immobilization as a Strategy for Improving Enzyme Properties-Application to Oxidoreductases. Molecules.

[16] Yuan Y., Shen J., Salmon S. (2023). Developing Enzyme Immobilization with Fibrous Membranes: Longevity and Characterization Considerations. Membranes.

[17] Xu K., Chen X., Zheng R., Zheng Y. (2020). Immobilization of Multi-Enzymes on Support Materials for Efficient Biocatalysis. Front. Bioeng. Biotechnol..

[18] Gupta K., Jana A.K., Kumar S., Maiti M. (2013). Immobilization of amyloglucosidase from SSF of Aspergillus niger by crosslinked enzyme aggregate onto magnetic nanoparticles using minimum amount of carrier and characterizations. J. Mol. Catal. B Enzym..

[19] Shan Z., Qianchun D., Ya L., Mingming Z., Chuyun W., Chang Z., Hu T., Fenghong H., Jie S. (2018). Novel amphiphilic polyvinylpyrrolidone functionalized silicone particles as carrier for low-cost lipase immobilization. R. Soc. Open Sci..

[20] Kahar U.M., Sani M.H., Chan K.-G., Goh K.M. (2016). Immobilization of α-Amylase from *Anoxybacillus* sp. SK3-4 on ReliZyme and Immobead Supports. Molecules.

[21] Singh R.K., Tiwari M.K., Singh R., Lee J.-K. (2013). From Protein Engineering to Immobilization: Promising Strategies for the Upgrade of Industrial Enzymes. Int. J. Mol. Sci..

[22] Wu H., Mu W. (2022). Application prospects and opportunities of inorganic nanomaterials for enzyme immobilization in the food-processing industry. Curr. Opin. Food Sci..

[23] Maghraby Y.R., El-Shabasy R.M., Ibrahim A.H., El-Said Azzazy H.M. (2023). Enzyme Immobilization Technologies and Industrial Applications. ACS Omega.

[24] Rodrigues R.C., Berenguer-Murcia Á., Carballares D., Morellon-Sterling R., Fernandez-Lafuente R. (2021). Stabilization of enzymes via immobilization: Multipoint covalent attachment and other stabilization strategies. Biotechnol. Adv..

[25] Almulaiky Y.Q., Aqlan F.M., Aldhahri M., Baeshen M., Khan T.J., Khan K.A., Afifi M., AL-Farga A., Warsi M.K., Alkhaled M. (2018). α-Amylase Immobilization on Amidoximated Acrylic Microfibres Activated by Cyanuric Chloride. R. Soc. Open Sci..

[26] Temoçin Z. (2014). Immobilization of *α*-amylase on reactive modified fiber and its application for continuous starch hydrolysis in a packed bed bioreactor. Starch–Stärke.

[27] Talebi M., Vaezifar S., Jafary F., Fazilati M., Motamedi S. (2016). Stability Improvement of Immobilized α-amylase using Nano Pore Zeolite. Iran. J. Biotechnol..

[28] Pereira S.E., Fernandes K.F., Ulhoa C.J. (2017). Immobilization of *Cryptococcus flavus* α-amylase on glass tubes and its application in starch hydrolysis. Starch–Stärke.

[29] Guzik U., Matos M.J., Pina A.S., Roque A. (2020). Rational design of affinity ligands for bioseparation. J. Chromatogr. A.

[30] Boudrant J., Woodley J.M., Fernandez-Lafuente R. (2020). Parameters necessary to define an immobilized enzyme preparation. Process. Biochem..

[31] Datta S., Christena L.R., Rajaram Y.R. (2013). Enzyme immobilization: An overview on techniques and support materials. 3 Biotech.

[32] Zucca P., Fernandez-Lafuente R., Sanjust E. (2016). Agarose and Its Derivatives as Supports for Enzyme Immobilization. Molecules.

[33] Azizi V., Mohammadi M., Rezaei Mokarram R., Sowti Khiabani M., Hamishehkar H. (2021). Immobilization of α-amylase on modified magnetic zeolite (MAZE) coated with carboxymethyl cellulose (CMC) composite and its properties. LWT.

[34] Kahraman M.V., Bayramoğlu G., Kayaman-Apohan N., Güngör A. (2007). α-Amylase immobilization on functionalized glass beads by covalent attachment. Food Chem..

[35] Silva F.S., Pio F.S., Ribeiro E.J., de Resende M.M. (2023). Immobilization of alpha-amylase (Termamyl® 2X) in Duolite® A-568 resin. Biocatal. Agric. Biotechnol..

[36] Migneault I., Dartiguenave C., Bertrand M.J., Waldron K.C. (2004). Glutaraldehyde: Behavior in aqueous solution, reaction with proteins, and application to enzyme crosslinking. BioTechniques.

[37] Serra I., Benucci I., Robescu M.S., Lombardelli C., Esti M., Calvio C., Pregnolato M., Terreni M., Bavaro T. (2019). Developing a Novel Enzyme Immobilization Process by Activation of Epoxy Carriers with Glucosamine for Pharmaceutical and Food Applications. Catalysts.

[38] Basso A., Serban S. (2019). Industrial applications of immobilized enzymes—A review. Mol. Catal..

[39] Takeuchi T., Kozu T., Watanabe S., Morita M., Shiratori K., Shibata I. (1978). Substrate specificity for pancreatic amylase. Gastroenterol. Jpn..

[40] Beyler-Çiğil A., Çakmakçı E., Danış Ö., Demir S., Kahraman M.V. (2013). Alpha-amylase Immobilization on Modified Polyimide Material. Chem. Eng. Trans..

[41] Mateo C., Palomo J.M., Fernandez-Lorente G., Guisan J.M., Fernandez-Lafuente R. (2007). Improvement of enzyme activity, stability and selectivity via immobilization techniques. Enzyme Microb. Technol..

[42] Zdarta J., Meyer A.S., Jesionowski T., Pinelo M.A. (2018). General Overview of Support Materials for Enzyme Immobilization: Characteristics, Properties, Practical Utility. Catalysts.

[43] Al-Najada A.R., Almulaiky Y.Q., Aldhahri M., El-Shishtawy R.M., Mohamed S.A., Baeshen M., Al-Farga A., Abdulaal W.H., Al-Harbi S.A. (2019). Immobilisation of α-amylase on activated amidrazone acrylic fabric: A new approach for the enhancement of enzyme stability and reusability. Sci. Rep..

[44] Jin W., Wang Z., Peng D., Shen W., Zhu Z., Cheng S., Li B., Huang Q. (2020). Effect of linear charge density of polysaccharides on interactions with α-amylase: Self-Assembling behavior and application in enzyme immobilization. Food Chem..

[45] Cipolatti E.P., Silva M.J.A., Klein M., Feddern V., Feltes M.M.C., Oliveira J.V., Ninow J.L., de Oliveira D. (2014). Current status and trends in enzymatic nano immobilization. J. Mol. Catal. B Enzym..

[46] Singh V., Rakshit K., Rathee S., Angmo S., Kaushal S., Garg P., Chung J.H., Sandhir R., Sangwan R.S., Singhal N. (2016). Metallic/bimetallic magnetic nanoparticle functionalization for immobilization of α-amylase for enhanced reusability in bio-catalytic processes. Bioresour. Technol..

[47] Mafakher L., Ahmadi Y., Khalili Fard J., Yazdansetad S., Rezaei Gomari S., Elyasi Far B. (2022). Alpha-Amylase Immobilization; Methods and Challenges. Pharm. Sci..

[48] Bié J., Sepodes B., Fernandes P.C.B., Ribeiro M.H.L. (2022). Enzyme Immobilization and Co-Immobilization: Main Framework, Advances and Some Applications. Processes.

[49] Gonçalves M., Amaral J.C., Lopes L.A., Fernandez-Lafuente R., Tardioli P.W. (2021). Stabilization and operational selectivity alteration of Lipozyme 435 by its coating with polyethyleneimine: Comparison of the biocatalyst performance in the synthesis of xylose fatty esters. Int. J. Biol. Macromol..

[50] Imam T.I., Marr P.C., Marr A.C. (2021). Enzyme entrapment, biocatalyst immobilization without covalent attachment. Green. Chem..

[51] Nisha S., Karthick S.A., Gobi N. (2012). A Review on Methods, Application and Properties of Immobilized Enzyme. Chem. Sci. Rev. Lett..

[52] Thangaraj B., Solomon P.R. (2019). Immobilization of lipases—A review. Part I: Enzyme immobilization. ChemBioEng. Rev..

[53] Bilal M., Iqbal H.M. (2019). Naturally-derived biopolymers: Potential platforms for enzyme immobilization. Int. J. Biol. Macromol..

[54] Arruda L.M.O., Vitolo M. (1999). Characterization of invertase entrapped into calcium alginate beads. Appl. Biochem. Biotechnol..

[55] Quiroga E., Illanes C.O., Ochoa N.A., Barberis S. (2011). Performance improvement of araujiain, a cystein phytoprotease, by immobilization within calcium alginate beads. Process. Biochem..

[56] Sundarram A., Murthy T.P.K. (2014). α-Amylase Production and Applications: A Review. Appl. Environ. Microbiol..

[57] Rodríguez V.B., Alameda E.J., Gallegos J.F.M., Requena A.R., López A.I.G. (2006). Enzymatic Hydrolysis of Soluble Starch with an α-Amylase from Bacillus licheniformis. Biotechnol. Progress.

[58] Ishikawa K., Matsui I., Honda K., Kobayashi S., Nakatani H. (1991). The pH dependence of the action pattern in porcine pancreatic alpha-amylase-catalyzed reaction for maltooligosaccharide substrates. Arch. Biochem. Biophys..

[59] Warren F.J., Zhang B., Waltzer G., Gidley M.J., Dhital S. (2015). The interplay of rmalpha-amylase and amyloglucosidase activities on the digestion of starch in in vitro enzymic systems. Carbohydr. Polym..

[60] Far B.E., Ahmadi Y., Khosroshahi A.Y., Dilmaghani A. (2020). Microbial Alpha-Amylase Production: Progress, Challenges and Perspectives. Adv. Pharm. Bull..

[61] Tiarsa E.R., Yandri Y., Suhartati T., Satria H., Irawan B., Hadi S. (2022). The Stability Improvement of *Aspergillus fumigatus α*-Amylase by Immobilization onto Chitin-Bentonite Hybrid. Biochem. Res. Int..

[62] Umit U., Melike Y.A. (2019). Immobilization and some application of α-amylase purified from *Rhizoctonia solani* AG-4 strain ZB-34. Turkish J. Biochem..

[63] Qin T., Liu Y., Zhao H., Xia X., Lei X. (2018). Cloning, expression, and characterization of a porcine pancreatic α-amylase in *Pichia pastoris*. Anim. Nutr..

[64] Robyt J.F., French D. (1967). Multiple attack hypothesis of α-amylase action: Action of porcine pancreatic, human salivary, and *aspergillus oryzae* α-amylases. Arch. Biochem. Biophys..

[65] Robyt J.F., French D. (1970). The action pattern of porcine pancreatic α-amylase in relationship to the substrate binding site of the enzyme. J. Biol. Chem..

[66] Breyer W.A., Matthews B.W. (2001). A structural basis for processivity. Protein Sci..

[67] Larson S.B., Day J.S., McPherson A. (2010). X-ray Crystallographic Analyses of Pig Pancreatic α-Amylase with Limit Dextrin, Oligosaccharide and α-Cyclodextrin. Biochemistry.

[68] Kyukiumar S., Chakravarty S., Nunes C.S., Kumar V. (2018). Amylases. Enzymes in Human and Animal Nutrition.

[69] Anitha Gopal B., Muralikrishna G. (2009). Porcine Pancreatic α-Amylase and its Isoforms: Purification and Kinetic Studies. Int. J. Food Prop..

[70] Fatoki O.A., Onilude A.A. (2022). Characterisation of alpha-amylase inhibitor from Streptomyces xinghaiensis AAI2 in solid substrate. Sci. Afr..

[71] Liao M., Liang G., Zhu J., Lu B., Peng X., Wang Y., Wei T., Zhou P., Huang B. (2019). Influence of Calcium Ions on the Thermal Characteristics of α-amylase from Thermophilic *Anoxybacillus* sp. GXS-BL. Protein Pept. Lett..

[72] Prejanò M., Alberto M.E., Russo N., Toscano M., Marino T. (2020). The Effects of the Metal Ion Substitution into the Active Site of Metalloenzymes: A Theoretical Insight on Some Selected Cases. Catalysts.

[73] Saha K., Maity S., Roy S., Pahan K., Pathak R., Majumdar S., Gupta S. (2014). Optimization of Amylase Production from *B. amyloliquefaciens* (MTCC 1270) Using Solid State Fermentation. Int. J. Microbiol..

[74] Hardwicke J.T., Hart J., Bell A., Duncan R., Thomas D.W., Moseley R. (2011). The effect of dextrin–rhEGF on the healing of full-thickness, excisional wounds in the (db/db) diabetic mouse. J. Control. Release.

[75] Hardwicke J., Ferguson E.L., Moseley R., Stephens P., Thomas D.W., Duncan R. (2008). Dextrin–rhEGF conjugates as bioresponsive nanomedicines for wound repair. J. Control. Release.

[76] Azzopardi E.A., Conlan R.S., Whitaker I.S. (2016). Polymer therapeutics in surgery: The next frontier. J. Interdiscip. Nanomed..

[77] Ritivoiu M.-E., Drăgoi C.M., Matei D., Stan I.V., Nicolae A.C., Craiu M., Dumitrescu I.-B., Ciolpan A.A. (2023). Current and Future Therapeutic Approaches of Exocrine Pancreatic Insufficiency in Children with Cystic Fibrosis in the Era of Personalized Medicine. Pharmaceutics.

[78] Iurciuc C.E., Savin A., Lungu C., Martin P., Popa M. (2016). Gellan. Food Applications. Cell. Chem. Technol..

[79] Iurciuc C.E., Lungu C., Martin P., Popa M. (2017). Gellan. Pharmaceutical, Medical and Cosmetic Applications. Cell. Chem. Technol..

[80] Chakraborty S., Jana S., Gandhi A., Sen K.K., Zhiang W., Kokare C. (2014). Gellan gum microspheres containing a novel α-amylase from marine *Nocardiopsis* sp. strain B2 for immobilization. Int. J. Biol. Macromol..

[81] Jadhav S.B., Singhal R.S. (2013). Screening of polysaccharides for preparation of α-amylase conjugate to enhance stability and storage life. Carbohydr. Polym..

[82] Iurciuc C.E., Alupei L., Savin A., Ibănescu C., Martin P., Popa M. (2016). Yeast cells immobilized in spherical gellan particles cross-linked with magnesium acetate. J. Biotechnol..

[83] Iurciuc C.E., Peptu C., Savin A., Atanase L.I., Souidi K., Mackenzie G., Martin P., Riess G., Popa M. (2017). Microencapsulation of Baker’s Yeast in Gellan Gum Beads Used in Repeated Cycles of Glucose Fermentation. Int. J. Polym. Sci..

[84] Iurciuc C.E., Savin A., Atanase L.I., Danu M., Martin P., Popa M. (2018). Encapsulation of Saccharomyces cerevisiae in hydrogel particles based gellan ionically cross-linked with zinc acetate. Powder Technol..

[85] Swami O.C., Shah N.J. (2017). Functional dyspepsia and the role of digestive enzymes supplement in its therapy. Int. J. Basic Clin. Pharmacol..

[86] Iurciuc-Tincu C.E., Atanase L.I., Ochiuz L., Jérôme C., Sol V., Martin P., Popa M. (2020). Curcumin-loaded polysaccharides-based complex particles obtained by polyelectrolyte complexation and ionic gelation. I-Particles obtaining and characterization. Int. J. Biol. Macromol..

[87] Oliveira H.M., Pinheiro A.Q., Fonseca A.J., Cabrita A.R., Maia M.R. (2019). Flexible and expeditious assay for quantitative monitoring of alpha-amylase and amyloglucosidase activities. MethodsX.

[88] Xiao Z., Storms R., Tsang A. (2006). A quantitative starch–iodine method for measuring alpha-amylase and glucoamylase activities. Anal. Biochem..

[89] Aksoy S., Tumturk H., Hasirci N. (1998). Stability of α-amylase immobilized on poly(methylmethacrylate-acrylic acid) microspheres. J. Biotechnol..

[90] Guo H., Tang Y., Yu Y., Xue L., Qian J.-Q. (2016). Covalent immobilization of *α*-amylase on magnetic particles as catalyst for hydrolysis of high-amylose starch. Int. J. Biol. Macromol..

[91] Sohrabi N., Rasouli N., Torkzadeh M. (2014). Enhanced stability and catalytic activity of immobilized α-amylase on modified Fe_3_O_4_ nanoparticles. Chem. Eng. J..

[92] Akhond M., Pashangeh K., Karbalaei-Heidari H.R., Absalan G. (2016). Efficient Immobilization of Porcine Pancreatic α-Amylase on Amino-Functionalized Magnetite Nanoparticles: Characterization and Stability Evaluation of the Immobilized Enzyme. Appl. Biochem. Biotechnol..

[93] Derkach S.R., Voron’ko N.G., Sokolan N.I., Kolotova D.S., Kuchina Y.A. (2020). Interactions between gelatin and sodium alginate: UV and FTIR studies. J. Dispers. Sci. Technol..

[94] Ernest V., Nirmala M.J., Gajalakshmi S., Mukherjee A., Chandrasekaran N. (2013). Biophysical Investigation of *α*-Amylase Conjugated Silver Nanoparticles Proves Structural Changes Besides Increasing Its Enzyme Activity. J. Bionanosci..

[95] Mikula K., Skrzypczak D., Ligas B., Witek-Krowiak A. (2019). Preparation of hydrogel composites using Ca^2+^ and Cu^2+^ ions as cross-linking agents. SN Appl. Sci..

[96] Charlet A., Lutz-Bueno V., Mezzenga R., Amstad E. (2021). Shape retaining self-healing metal-coordinated hydrogels. Nanoscale.

[97] Fukuda H. (1980). Polyelectrolyte Complexes of Chitosan with Sodium Carboxymethylcellulose. Bull. Chem. Soc. Jpn..

[98] Dellali M., Iurciuc C.E., Savin C.L., Spahis N., Djennad M., Popa M. (2021). Hydrogel Films Based on Chitosan and Oxidized Carboxymethylcellulose Optimized for the Controlled Release of Curcumin with Applications in Treating Dermatological Conditions. Molecules.

[99] Derkach S.R., Kuchina Y.A. (2022). Intermolecular Interactions in the Formation of Polysaccharide-Gelatin Complexes: A Spectroscopic Study. Polymers.

[100] Kanyuck K.M., Mills T.B., Norton I.T., Norton-Welch A.B. (2021). Swelling of high acyl gellan gum hydrogel: Characterization of network strengthening and slower release. Carbohydr. Polym..

[101] Picone C.S.F., Cunha R.L. (2011). Influence of pH on formation and properties of gellan gels. Carbohydr. Polym..

[102] Santos T.P., Cunha R.L. (2018). Role of process variables on the formation and in vitro digestion of gellan gels. Carbohydr. Polym..

[103] Talekar S., Chavare S. (2012). Optimization of Immobilization of α-Amylase in Alginate Gel and Its Comparative Biochemical Studies with Free α-Amylase. Recent. Res. Sci. Technol..

[104] Zhang C., Grossier R., Candoni N., Veesler S. (2021). Preparation of alginate hydrogel microparticles by gelation introducing cross-linkers using droplet-based microfluidics: A review of methods. Biomater. Res..

[105] Lyu X., Gonzalez R., Horton A., Li T. (2021). Immobilization of Enzymes by Polymeric Materials. Catalysts.

[106] Yadav J.K., Prakash V. (2011). Stabilization of α-Amylase, the Key Enzyme in Carbohydrates Properties Alterations, at Low pH. Int. J. Food Prop..

[107] Ahmed N.E., El Shamy A.R., Awad H.M. (2020). Optimization and immobilization of amylase produced by *Aspergillus terreus* using pomegranate peel waste. Bull. Natl. Res. Cent..

[108] El-Banna T.E., Abd-Aziz A.A., Abou-Dobara M.I., Reham I.I. (2007). Production and Immobilization of α-Amylase from *Bacillus subtilis*. Pak. J. Biol. Sci..

[109] Hemanchi H.K., Sanjay N.P. (2019). Immobilization of α-Amylase by Entrapment Method and Its Comparative Study with Free α-Amylase. Int. J. Res. Appl. Sci. Eng. Technol..

[110] Udema I.I. (2016). Calcium Ion Binding Characteristics of Porcine Pancreatic AlphaAmylase outside Active Site Domain and Implications: Theory and Experimentation. Adv. Res..

[111] Castañeda Ruiz A.J., Shetab Boushehri M.A., Phan T., Carle S., Garidel P., Buske J., Lamprecht A. (2022). Alternative Excipients for Protein Stabilization in Protein Therapeutics: Overcoming the Limitations of Polysorbates. Pharmaceutics.

[112] Singh V., Kumar P. (2011). Carboxymethyl tamarind gum–silica nanohybrids for effective immobilization of amylase. J. Mol. Catal. B Enzym..

[113] Sinha R., Khare S.K. (2014). Protective role of salt in catalysis and maintaining structure of halophilic proteins against denaturation. Front. Microbiol..

[114] Dutta T.K., Jana M., Pahari P.R., Bhattacharya T. (2006). The Effect of Temperature, pH, and Salt on Amylase in *Heliodiaptomus viduus* (Gurney) (Crustacea: Copepoda: Calanoida). Turk. Zool. Derg..

[115] Dey G., Singh B., Banerjee R. (2003). Immobilization of α-Amylase Produced by *Bacillus circulans* GRS 313. Braz. Arch. Biol. Technol..

[116] Qiu H., Xu C., Huang X., Ding Y., Qu Y., Gao P. (2009). Immobilization of Laccase on Nanoporous Gold: Comparative Studies on the Immobilization Strategies and the Particle Size Effects. J. Phys. Chem. C.

[117] Defaei M., Taheri-Kafrani A., Miroliaei M., Yaghmaei P. (2018). Improvement of stability and reusability of α-amylase immobilized on naringin functionalized magnetic nanoparticles: A robust nanobiocatalyst. Int. J. Biol. Macromol..

[118] Yoo Y.J., Hong J., Hatch R.T. (1987). Comparison of alpha-amylase activities from different assay methods. Biotechnol. Bioeng..

[119] Lowry O.H., Rosebrough N.J., Farr A.L., Randall R.J. (1951). Protein Measurement with the Folin Phenol Reagent. J. Biol. Chem..

[120] Al-Mayah A.M.R. (2012). Simulation of Enzyme Catalysis in Calcium Alginate Beads. Enzyme Res..

