# Supplementary material for: An Accessible Method to Improve the Stability and Reusability of Porcine Pancreatic α-Amylase via Immobilization in Gellan-Based Hydrogel Particles Obtained by Ionic Cross-Linking with Mg2+ Ions
Source: Molecules. 2023 Jun 11;28(12):4695. doi: 10.3390/molecules28124695 (PMC10302431; doi:10.3390/molecules28124695)
Supplement: Supplementary file 1 [file molecules-28-04695-s001.zip › molecules-2341679-supplementary.pdf]

Supplementary materials

The article's title: An Accessible Method to Improve the Stability and Reusability of Porcine Pancreatic α-Amylase via Immobilization in Gellan-Based Hydrogel Particles Obtained by Ionic Cross-Linking with Mg^{2+} Ions.

Authors: Camelia Elena Tincu (Iurciuc), Brahim Bouhadiba, Leonard Ionut Atanase, Corneliu Sergiu Stan, Marcel Popa*, Lăcrămioara Ochiuz

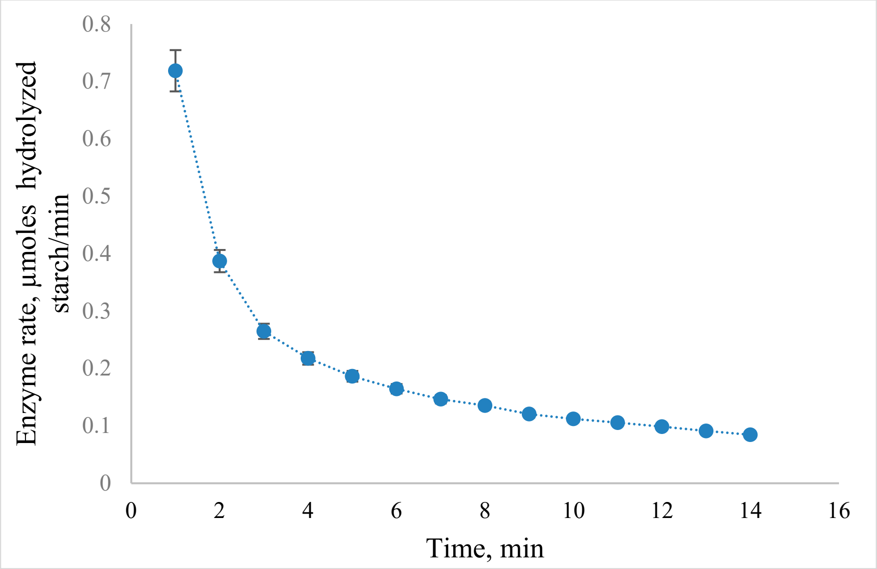

Figure S1. Variation of starch hydrolysis rate over time using a constant concentration of free enzyme and starch.

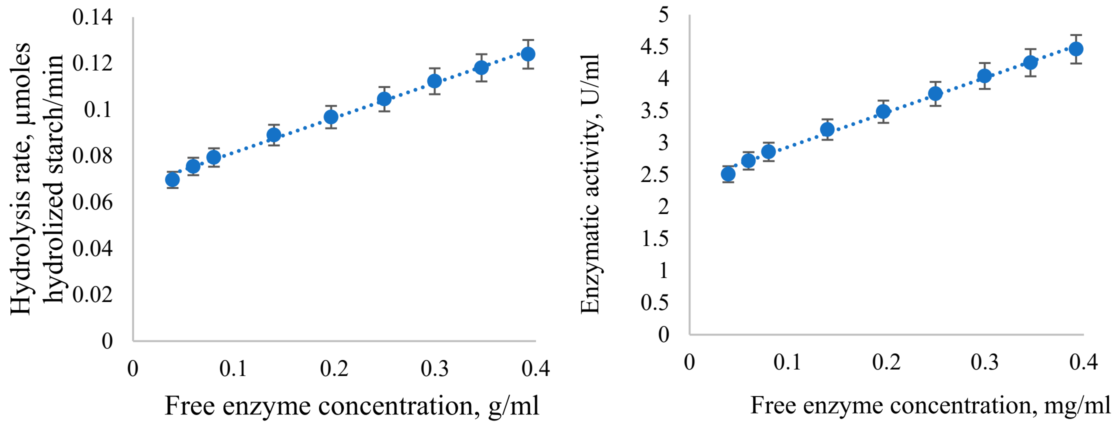

(a) (b)

Figure S2. Variation of starch hydrolysis rate (expressed as µmoles of hydrolyzed starch/min)-(a) and enzymatic activity (b) using different concentrations for free enzyme and a constant starch concentration.

Table S1. Determination of the enzymatic activity for particles in which *α*-amylase was encapsulated at different temperatures.

Sample	Encapsulation temperature, ^{0}C	Enzyme activity, U/g particles ml
A3-1	40	3.59±0.4
A3-2	50	4.13±0.55
A3-3	60	4.45±0.36

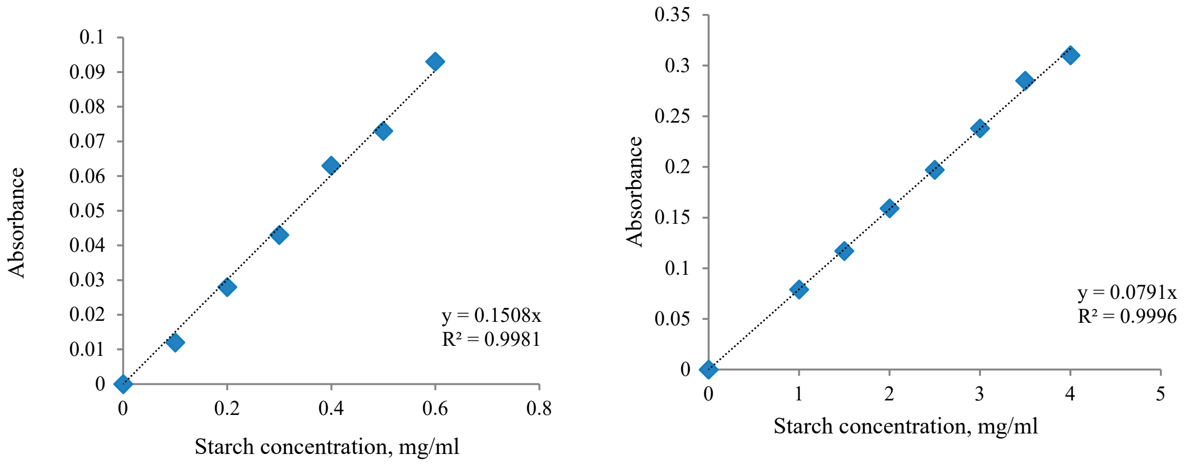

(a) (b)

Figure S3. Calibration curves for starch using (a) concentrations less than 1 mg/ml and (b) concentrations up to 4 mg/ml.

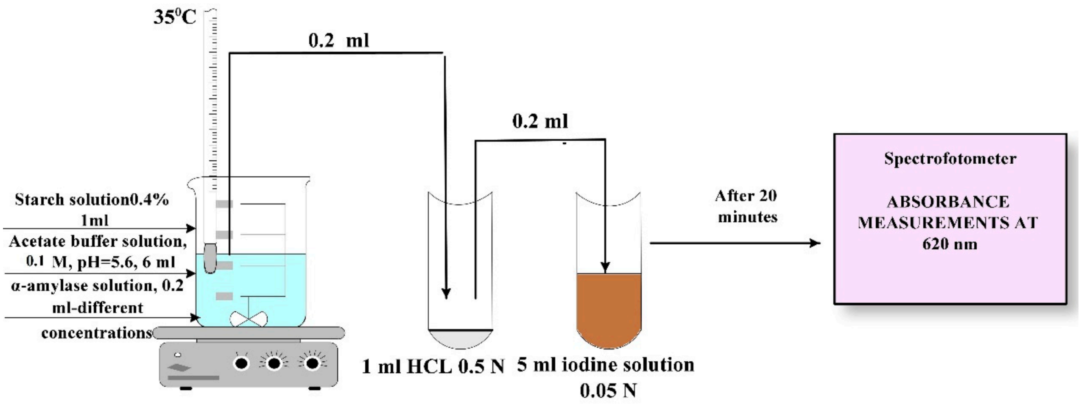

Figure S4. Schematic presentation of the working method for determining the activity of free or immobilized α-amylase.

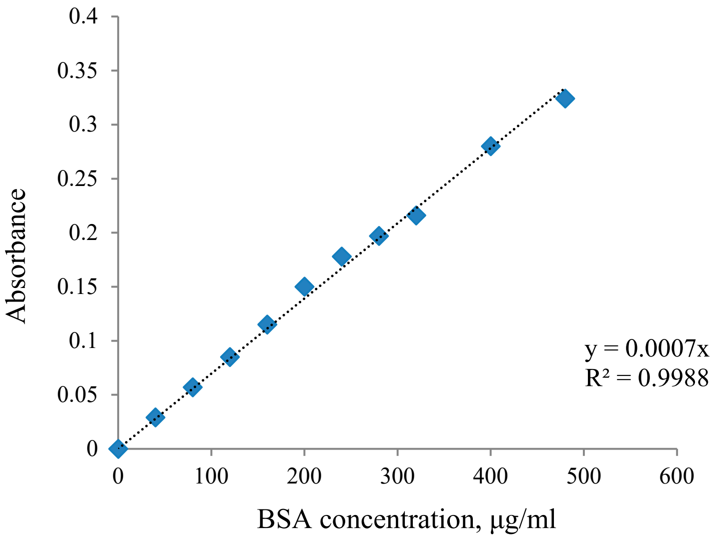

Figure S5. Bovine serum albumin (BSA) calibration curve.
